# Tumour‐microenvironment‐responsive Na_2_S_2_O_8_ nanocrystals encapsulated in hollow organosilica–metal–phenolic networks for cycling persistent tumour‐dynamic therapy

**DOI:** 10.1002/EXP.20230054

**Published:** 2023-11-14

**Authors:** Yang Li, Jinyan Lin, Yueyang He, Kaiyuan Wang, Cailin Huang, Ruifeng Zhang, Xiaolong Liu

**Affiliations:** ^1^ The United Innovation of Mengchao Hepatobiliary Technology Key Laboratory of Fujian Province Mengchao Hepatobiliary Hospital of Fujian Medical University Fuzhou People's Republic of China; ^2^ CAS Key Laboratory of Design and Assembly of Functional Nanostructures, Fujian Institute of Research on the Structure of Matter Chinese Academy of Sciences Fuzhou People's Republic of China; ^3^ Department of Translational Medicine and Xiamen Key Laboratory of Rare Earth Photoelectric Functional Materials, Xiamen Institute of Rare‐Earth Materials, Haixi Institute Chinese Academy of Sciences Xiamen People's Republic of China; ^4^ Xiang'an Hospital of Xiamen University, School of Medicine Xiamen University Xiamen People's Republic of China; ^5^ Departments of Diagnostic Radiology, Surgery, Chemical and Biomolecular Engineering, and Biomedical Engineering, Yong Loo Lin School of Medicine and Faculty of Engineering National University of Singapore Singapore Singapore; ^6^ Department of Pharmaceutics, Wuya College of Innovation Shenyang Pharmaceutical University Shenyang People's Republic of China

**Keywords:** cascade‐responsive, dual cycling amplification of •SO_4_^−^/•OH, tumour microenvironment

## Abstract

Traditional tumour‐dynamic therapy still inevitably faces the critical challenge of limited reactive oxygen species (ROS)‐generating efficiency due to tumour hypoxia, extreme pH condition for Fenton reaction, and unsustainable mono‐catalytic reaction. To fight against these issues, we skilfully develop a tumour‐microenvironment‐driven yolk‐shell nanoreactor to realize the high‐efficiency persistent dynamic therapy via cascade‐responsive dual cycling amplification of •SO_4_^−^/•OH radicals. The nanoreactor with an ultrahigh payload of free radical initiator is designed by encapsulating the Na_2_S_2_O_8_ nanocrystals into hollow tetra‐sulphide‐introduced mesoporous silica (HTSMS) and afterward enclosed by epigallocatechin gallate (EG)‐Fe(II) cross‐linking. Within the tumour microenvironment, the intracellular glutathione (GSH) can trigger the tetra‐sulphide cleavage of nanoreactors to explosively release Na^+^/S_2_O_8_^2^ **^−^**/Fe^2+^ and EG. Then a sequence of cascade reactions will be activated to efficiently generate •SO_4_^−^ (Fe^2+^‐catalyzed S_2_O_8_^2^ **^−^** oxidation), proton (•SO_4_^−^‐catalyzed H_2_O decomposition), and •OH (proton‐intensified Fenton oxidation). Synchronously, the oxidation‐generated Fe^3+^ will be in turn recovered into Fe^2+^ by excessive EG to circularly amplify •SO_4_^−^/•OH radicals. The nanoreactors can also disrupt the intracellular osmolarity homeostasis by Na^+^ overload and weaken the ROS‐scavenging systems by GSH exhaustion to further amplify oxidative stress. Our yolk–shell nanoreactors can efficiently eradicate tumours via multiple oxidative stress amplification, which will provide a perspective to explore dynamic therapy.

## Graphical abstract

Traditional tumour‐dynamic therapy still inevitably faces the critical challenge of limited reative oxygen species (ROS)‐generating efficiency due to tumour hypoxia, strict Fenton reaction conditions, and un‐sustainable mono‐catalytic reaction. To fight against these issues, a tumour–microenvironment‐responsive yolk–shell nanoreactor is developed to realize the high‐efficiency, selective, and sustainable dynamic therapy via cascade‐responsive dual cycling amplification of •SO_4_^−^/•OH radicals.

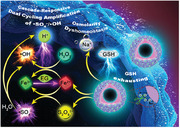

## 1 INTRODUCTION

Dynamic therapy (e.g. photodynamic therapy, sonodynamic therapy, chemodynamic therapy, and enzyme dynamic therapy) has attracted widespread attention for its non‐invasiveness and tumour‐specificity, which have presented tremendous potential in cancer therapy.^[^ ^1^, ^2^ ^]^ However, the reactive oxygen species (ROS)‐generating efficiency is extremely restricted by inadequate tumoral O_2_ levels and rigorous Fenton reaction conditions (pH 3–4).^[^ ^3^, ^4^, ^5^ ^]^ It is urgent to develop a novel tumour‐dynamic therapy with efficient ROS production capacity. •SO_4_^−^, a newly reported ROS (redox potential: 2.5–3.1 V; half‐life: 30 µs) has been demonstrated to be more effective in a wide range of pH values compared with •OH (redox potential: 1.9–2.7 V; shorter half‐life: <1 µs).^[^ ^6^, ^7^ ^]^ It has been indicated that the •SO_4_^−^ radical could be efficaciously generated through the oxidation of S_2_O_8_^2−^ or HSO_5_^−^ ions by transition‐metal ions (Co^2+^, Mn^2+^, Fe^2+^, etc.), which was frequently used in the organic pollutant degradation of wastewater.^[^ ^8^, ^9^, ^10^, ^11^ ^]^ Notably, this •SO_4_^−^ generation process is independent of pH value and O_2_ level.^[^ ^5^, ^8^, ^9^, ^10^, ^11^ ^]^ Thereby, this environmental remediation technology inspires us to develop a high‐efficiency •SO_4_^−^ generator to resolve the dilemma of traditional tumour‐dynamic therapy.

Nevertheless, it is hard to systemically deliver the various types of ions to tumours owing to rapid blood clearance, dose‐limiting systemic toxicity, and poor tumour selectivity.^[^ ^12^, ^13^, ^14^, ^15^ ^]^ In addition, due to the high water solubility of these ions, it is generally difficult to adequately load them within the nanomaterials.^[^ ^16^, ^17^, ^18^ ^]^ Hence, it is a challenging task to fabricate the S_2_O_8_^2−^ or HSO_5_^−^‐encapsulated nanoparticles with ultrahigh ion payload. Inspiringly, the cavity‐confined effect plays an essential role in the controlled nucleation, growth, and stabilization of cargos within the nanomaterial.^[^ ^19^, ^20^, ^21^ ^]^ One would wonder whether it is possible to load the Na_2_S_2_O_8_ nanocrystals within the interior cavity of a tumour‐microenvironment‐responsive hollow nanomaterial via such a cavity‐confined effect to realize the extremely high payload of S_2_O_8_^2−^ ions and precise delivery. Additionally, as a relatively safe transition‐metal ion, the endogenous Fe^2+^ is too limited to effectively catalyze the S_2_O_8_^2−^ ions to produce sufficient •SO_4_^−^ for tumour therapy.^[^ ^22^, ^23^ ^]^ Thereby, it is necessary to transport appropriate amounts of exogenous Fe^2+^ to the tumour sites. Nevertheless, the therapeutic effect of single free radical treatment is still limited due to its unsustainability. It is essential to develop a •SO_4_^−^ radical‐based therapeutic mode with sustainable generation of multiple free radicals. Interestingly, the •SO_4_^−^ radical can catalyze the decomposition of H_2_O to generate the proton,^[^ ^10^, ^11^ ^]^ which will provide a favourable condition for Fenton chemistry, to significantly accelerate the production of •OH. As the oxidation reaction proceeds, the catalytic efficiency will gradually decline due to the continuous consumption of Fe^2+^. Thereby, it is necessary to develop a •SO_4_ **^−^**/•OH generator that can replenish the Fe^2+^ within the tumour microenvironment, which will consistently keep the dynamic therapeutic efficiency.

**SCHEME 1:**
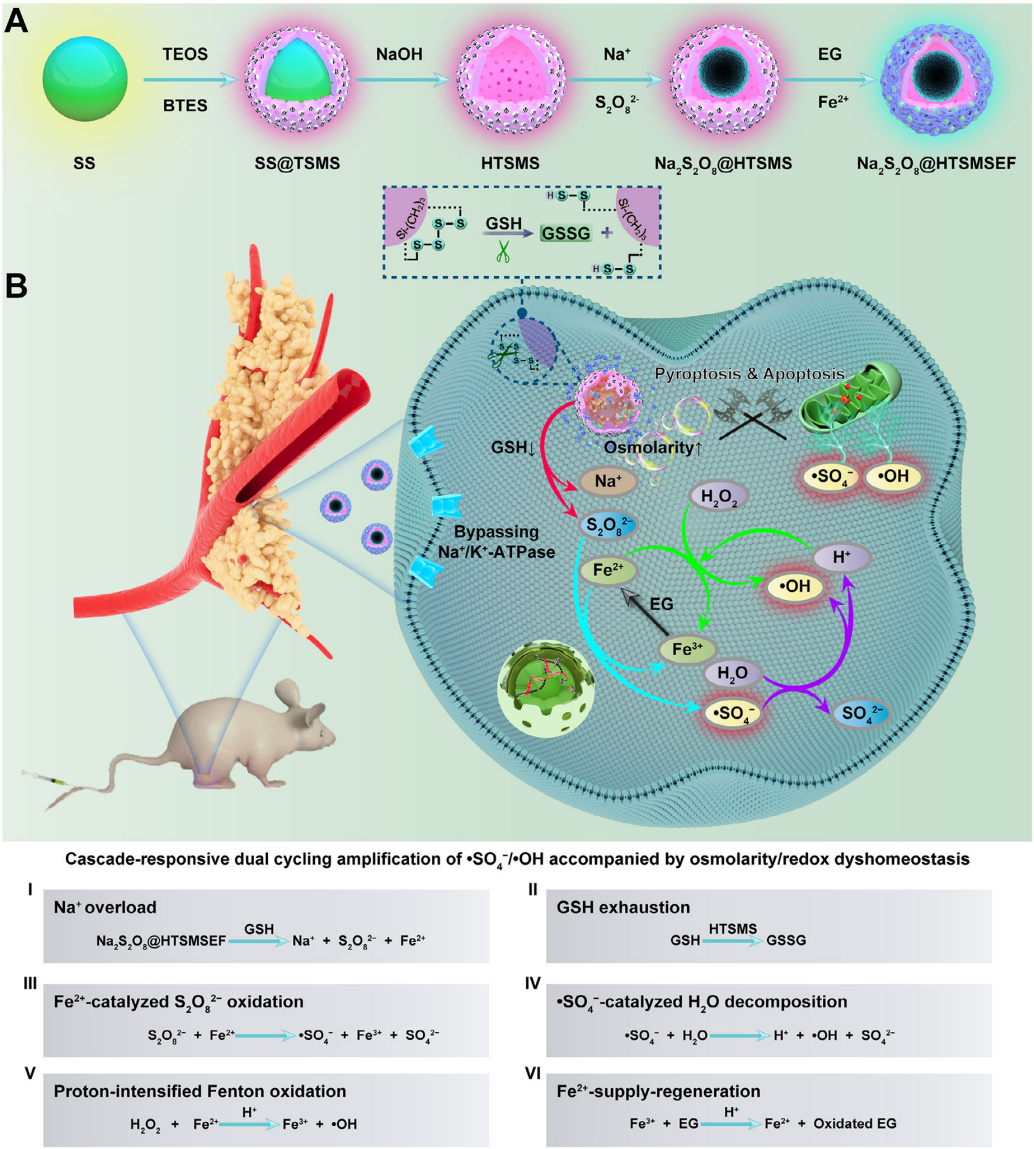
Preparation and working mechanism of tumor‐microenvironment‐driven yolk‐shell nanoreactors. (A) Schematic illustration of the preparation of nanoreactors. (B) Schematic illustration of the working mechanism of nanoreactors. A novel tumour‐microenvironment‐driven “metal‐phenolic network”‐cloaked hollow tetra‐sulphide‐introduced mesoporous organosilica is designed to temporospatially control the delivery of Na_2_S_2_O_8_ nanocrystals and sequential release the Na^+^/S_2_O_8_^2−^/Fe^2+^ and EG, which could synergistically improve the therapeutic efficiency via cascade‐responsive dual cycling amplification of •SO_4_^−^/•OH radicals accompanied by osmolarity/redox dyshomeostasis.

We herein develop a tumour‐microenvironment‐driven yolk–shell ROS nanoreactor with an ultrahigh payload of free radical initiator for high‐efficiency persistent dynamic therapy via cascade‐responsive dual cycling amplification of •SO_4_ **^−^**/•OH radicals (Scheme 1). The Na_2_S_2_O_8_ nanocrystals are first encapsulated into the interior of hollow tetra‐sulphide‐introduced mesoporous silica (HTSMS) through the cavity‐confined effect. Then, the Na_2_S_2_O_8_‐encapsulated HTSMS (denoted as Na_2_S_2_O_8_@HTSMS) is further enclosed by EG‐Fe(II) crosslinking (denoted as Na_2_S_2_O_8_@HTSMSEF) to avoid premature leakage during circulation and evoke a sequence of cascade reactions within the tumour microenvironment. Once accumulated in the tumours, the nanoreactors will respond to the intracellular overproduced glutathione (GSH) with shell erosion/degradation, triggering the burst release of Na^+^/S_2_O_8_^2−^/Fe^2+^ and EG. The S_2_O_8_^2−^ can be effectively catalyzed by the Fe^2+^ to transform into •SO_4_^−^. Significantly, the •SO_4_^−^ can catalyze the H_2_O decomposition to generate the proton to promote the Fenton oxidation of H_2_O_2_, so as to generate a high level of •OH. More importantly, the EG can be arranged to restore the oxidation‐generated Fe^3+^ into Fe^2+^ for cycling amplification of •SO_4_ **^−^**/•OH. In addition, the nanoreactors can also induce osmolarity dyshomeostasis by overloaded Na^+^ and weaken the ROS‐scavenging systems by GSH‐exhausting tetra‐sulphide framework to further intensify oxidative stress. Our tumour–microenvironment‐responsive yolk‐shell nanoreactors with multiple oxidative stress amplification will be a new therapeutic complementary to traditional dynamic therapy.

## 2 RESULTS AND DISCUSSION

### 2.1 Preparation and characterization of Na_2_S_2_O_8_@HTSMSEF

**FIGURE 1:**
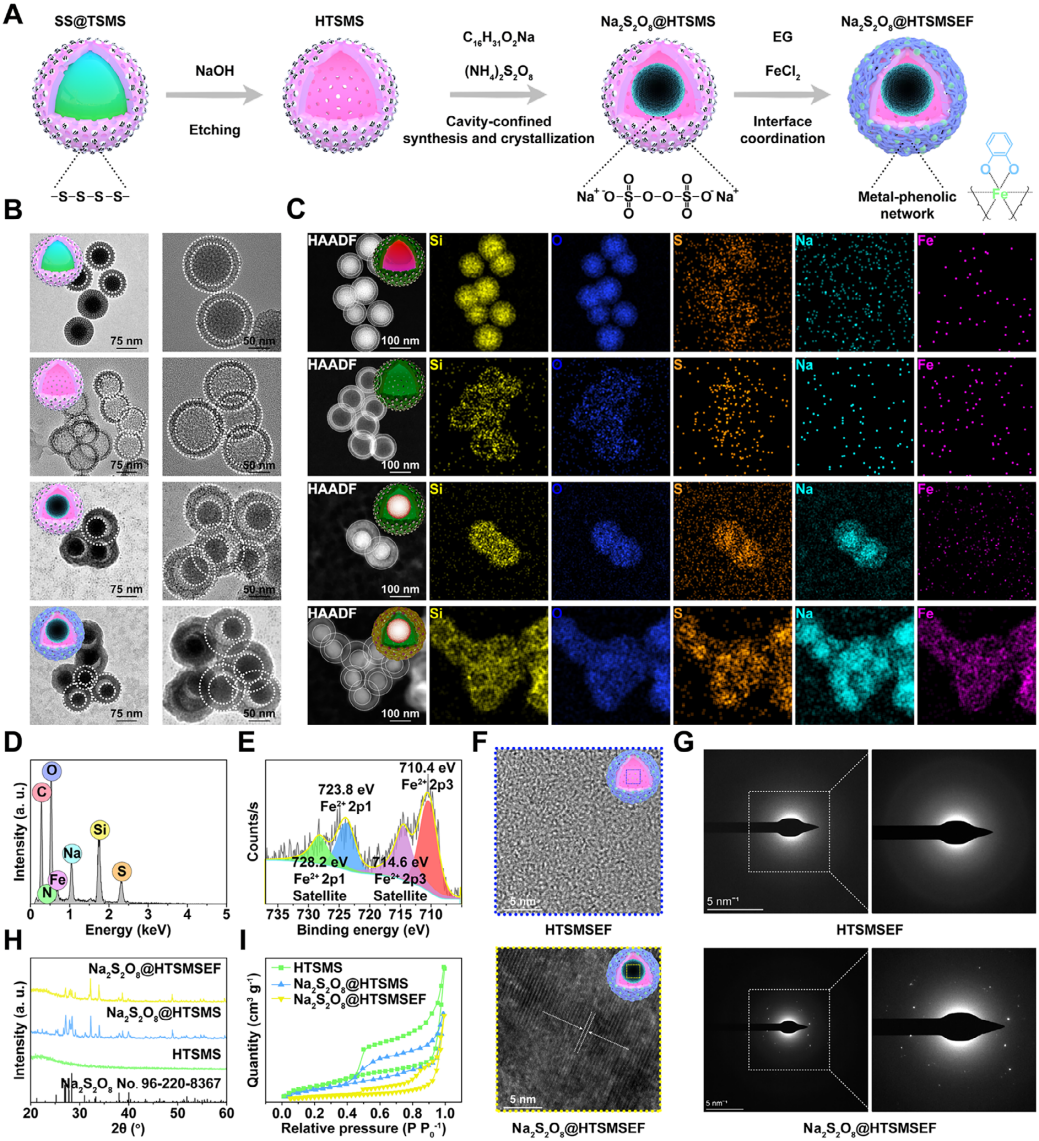
Preparation of Na_2_S_2_O_8_@HTSMSEF. (A) Illustration of preparation route of Na_2_S_2_O_8_@HTSMSEF. (B) TEM images of SS@TSMS, HTSMS, Na_2_S_2_O_8_@HTSMS, and Na_2_S_2_O_8_@HTSMSEF. (C) HAADF‐TEM images and EDX element mapping images of SS@TSMS, HTSMS, Na_2_S_2_O_8_@HTSMS, and Na_2_S_2_O_8_@HTSMSEF. (D) EDX spectrum of Na_2_S_2_O_8_@HTSMSEF in selected yolk–shell area. (E) XPS spectrum of Fe2p of Na_2_S_2_O_8_@HTSMSEF. (F) HRTEM images and (G) SAED patterns of HTSMSEF and Na_2_S_2_O_8_@HTSMSEF. (H) XRD patterns of HTSMS, Na_2_S_2_O_8_@HTSMSF, and Na_2_S_2_O_8_@HTSMSEF. (I) N_2_ adsorption‐desorption isotherms of HTSMS, Na_2_S_2_O_8_@HTSMSF, and Na_2_S_2_O_8_@HTSMSEF.

The hollow tetra‐sulphide‐introduced mesoporous silica (HTSMS) was fabricated via the etching of core–shell solid silica@tetra‐sulphide‐introduced mesoporous silica (SS@TSMS) (Figure 1A). During the fabrication of SS@TSMS, the typical solid silica (SS) nanoparticles^[^ ^24^ ^]^ (Figure S1) were utilized as a core, and cetyltrimethylammonium bromide (CTAB) was utilized to guide the mesoporous structural formation on the surface of SS. Noticeably, through a “chemical homology” principle, the SS@TSMS nanoparticles were fabricated via co‐condensation of tetraethyl orthosilicate (TEOS) with bis[3‐(triethoxysilyl)propyl] tetra‐sulphide (BTES) onto the SS surface.^[^ ^25^ ^]^ Afterward, sodium hydroxide was utilized to remove the core by etching to produce the HTSMS nanoparticles.

The HTSMS‐wrapped Na_2_S_2_O_8_ nanocrystals (Na_2_S_2_O_8_@HTSMS) were fabricated via a cavity‐confined synthesis and crystallization approach followed by an interface crosslinking approach. First, we prepared the Na_2_S_2_O_8_@HTSMS by adding C_15_H_31_COONa and HTSMS within the *n*‐hexane/ethanol/oleylamine solvent and successive adding of (NH_4_)_2_S_2_O_8_. It was noteworthy that Na_2_S_2_O_8_ molecules were in situ chemically synthesized to initiate their nucleation and growth within the cavity‐confined interior of HTSMS. Then, we prepared the Na_2_S_2_O_8_@HTSMSEF by adding the epigallocatechin gallate (EG) onto the Na_2_S_2_O_8_@HTSMS via high‐affinity interaction between catechol moiety^[^ ^26^, ^27^, ^28^ ^]^ and inorganic surface and subsequent adding of Fe^2+^ by robust metal–catechol interface coordination interaction.^[^ ^29^, ^30^ ^]^ The morphologies and surface charge of SS@TSMS, HTSMS, Na_2_S_2_O_8_@HTSMS, and Na_2_S_2_O_8_@HTSMSEF were investigated by transmission electron microscope (TEM) images and zeta potential analyzer (Figure 1B and Figures S2–S4). The Na_2_S_2_O_8_@HTSMS and Na_2_S_2_O_8_@HTSMSEF presented a uniform yolk‐shell structure with an average size of ≈125 nm. The energy dispersive X‐ray (EDX) elemental mapping images and EDX spectrum demonstrated the co‐existence of Na, S, O, Si, and Fe within the Na_2_S_2_O_8_@HTSMSEF (Figure 1C,D, and Figure S5). The X‐ray photoelectron spectroscopy (XPS) spectrum of Fe2p exhibited four peaks at 710.4, 714.6, 723.8, and 728.2 eV, which were assigned to the binding energies of Fe^2+^2p3, Fe^2+^2p3 satellite, Fe^2+^2p1, and Fe^2+^2p1 satellite, respectively, proving that Fe element existed in a bivalence state (Figure 1E and Figures S6–S8). The high‐resolution TEM (HRTEM) image testified the crystalline structure of Na_2_S_2_O_8_@HTSMSEF with ordered lattice fringes (Figure 1F and Figure S9). The selected area electron diffraction (SAED) result also indicated the diffuse ring characteristic of crystal with bright spots (Figure 1G). The X‐ray diffraction (XRD) patterns implied the crystal characteristic of Na_2_S_2_O_8_@HTSMSEF with a centrosymmetric space group P‐1 (Figure 1H and Figure S10), which was in line with the standard card of Na_2_S_2_O_8_ (Crystallography Open Database No. 96‐220‐8367). The N_2_ adsorption/desorption isotherms proved the well‐organized mesoporous characteristic of HTSMS (Figure 1I). In addition, the specific surface area by using Brunauer–Emmett–Teller (BET) method was significantly decreased in the Na_2_S_2_O_8_@HTSMSEF in comparison with those in the HTSMS and Na_2_S_2_O_8_@HTSMS (Figure S11), indicating the successful encapsulation of Na_2_S_2_O_8_ nanocrystals within the cavity of HTSMS and subsequent interface crosslinking of EG‐Fe(II) on the surface of Na_2_S_2_O_8_@HTSMS (Figures S12–S14). By using TEM dynamic observation, it was found that the nanoreactors were kept intact with the yolk–shell structure under different physiological conditions (Figures S15 and S16). The result indicated the excellent physiological stability of Na_2_S_2_O_8_@HTSMSEF, which was likely explained by the presence of EG‐Fe(II) as a protective layer.

### 2.2 GSH‐responsive dynamics

**FIGURE 2:**
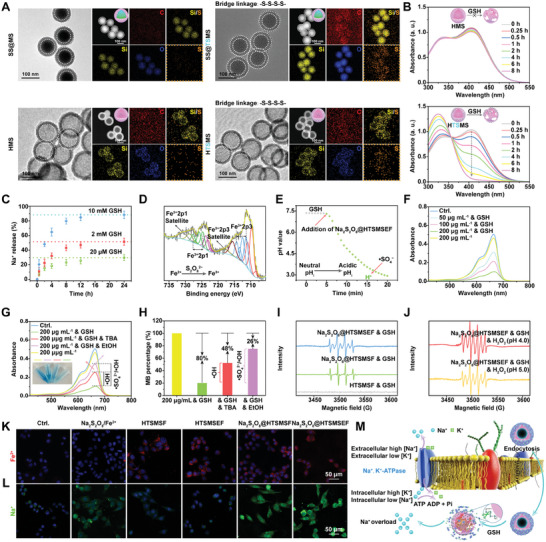
Characterization and intracellular Na^+^/Fe^2+^ delivery of Na_2_S_2_O_8_@HTSMSEF. (A) TEM, HAADF‐TEM, and EDX elemental mapping images of SS@MS, SS@TSMS, HMS, and HTSMS (EDX elemental mapping images of SS@TSMS and HTSMS originated from Figure 1C). (B) UV–vis spectra of DTNB in HMS and HTSMS‐pretreated GSH. (C) Release kinetics of Na^+^ from Na_2_S_2_O_8_@HTSMSEF in potassium phosphate buffer with different GSH concentrations using Na^+^ electrode (*n* = 3). (D) XPS spectra of Fe2p of Na_2_S_2_O_8_@HTSMSEF after GSH treatment, indicative of valence state transition of Fe element. (E) pH value changes of PBS buffer with GSH (10 mm) from neutrality to acidity after the addition of Na_2_S_2_O_8_@HTSMSEF using pH microelectrode. Inset: Illustration of intracellular microenvironment reshaping to intensify the Fenton oxidation to elevate the •SO_4_^−^/•OH generation efficiency. (F) UV–vis spectra of MB after the addition of GSH‐pretreated Na_2_S_2_O_8_@HTSMSEF, indicative of •SO_4_^−^/•OH generation ability. (G) UV–vis spectra and (H) percentage analysis of MB in Na_2_S_2_O_8_@HTSMSEF pretreated with GSH and treated with ROS scavenger (TBA: •OH scavenger; EtOH: •SO_4_^−^/•OH scavenger). (I) ESR spectra of both •SO_4_^−^/ and •OH in Na_2_S_2_O_8_@HTSMSEF after GSH treatment. (J) ESR spectra of both •SO_4_^−^/ and •OH in Na_2_S_2_O_8_@HTSMSEF after different treatments. (K,L) Confocal images of different formulations‐treated HepG2 cells for 4 h and stained with (K) FerroOrange (Fe^2+^ indicator)/(L) SBFI (Na^+^ indicator), indicative of intracellular Fe^2+^ and Na^+^ level. (M) Schematic illustration of endocytosis of HTSMSF‐wrapped Na_2_S_2_O_8_ nanocrystals into tumour cells followed by intracellular GSH‐triggered shell degradation. Due to the reverse osmosis gradient across the cellular membrane, a large amount of released Na^+^ would be restricted inside the cells without being effluxed to circumvent Na^+^, K^+^‐ATPase across the cellular membrane and thus cause the osmolarity surge.

To explore the GSH‐depleting ability of HTSMS, we selected the GSH‐irresponsive HMS as a control. First, EDX elemental mapping images proved the presence of tetra‐sulphide bridges within the HTSMS as evidenced by the enrichment of sulphur element (Figure 2A). Then we used 5, 5′‐dithiobis (2‐nitrobenzoic acid) (DTNB, which absorption peak was 325 nm) to test the unreacted GSH (DTNB could react with the sulfhydryl group of GSH to generate yellow TNB, which absorption peak was 412 nm) after adding GSH into HTSMS for different times. As presented in Figure 2B and Figure S17, with the prolonged incubation time, the ultraviolet (UV)–visible absorbance of TNB significantly declined in HTSMS‐pretreated GSH rather than in HMS‐pretreated GSH, implying the GSH‐exhausting functionality of HTSMS.

To further investigate the GSH‐responsive degradability of Na_2_S_2_O_8_@HTSMSEF, they were further introduced into potassium phosphate buffer with different GSH concentrations for various incubation times. By using Na^+^ electrode, the Na_2_S_2_O_8_@HTSMSEF upon various GSH concentrations exhibited GSH‐responsive Na^+^ release kinetics (Figure 2C). The results indicated adequate GSH‐responsive degradability for cargo release. Through the XPS analysis, the Fe^3+^2p3, Fe^3+^2p1, Fe^3+^2p3 satellite, and Fe^3+^2p1 satellite peaks were newly generated in Fe2p spectra after adding GSH into Na_2_S_2_O_8_@HTSMSEF (Figure 2D and Figure S18), implying that the released S_2_O_8_^2−^ could transform Fe^2+^ into Fe^3+^ by oxidation. The simultaneously generated •SO_4_^−^ would catalyze the decomposition of H_2_O to generate •OH and proton, and the latter was conducive to increasing the acidity to promote Fenton oxidation and EG activation. Interestingly, the PBS buffer with GSH (10 mM) exhibited a significant decline in pH values after Na_2_S_2_O_8_@HTSMSEF treatment (Figure 2E), indicating the successful acidification reshaping by •SO_4_^−^‐catalyzed H_2_O decomposition. It was expected that the morphological integrity could be kept to avoid the severe premature release of Na^+^/S_2_O_8_^2−^ in blood, but would be degraded to release the Na^+^/S_2_O_8_^2−^ ions after internalization into tumour cells for intracellular microenvironment reshaping.

### 2.3 •SO_4_^−^/•OH generation ability

To explore the free radical generation potential of Na_2_S_2_O_8_@HTSMSEF, we detected the degradability of methylene blue (MB) after adding various concentrations of Na_2_S_2_O_8_@HTSMSEF pretreated with GSH. As presented in Figure 2F, the degradability of MB was dependent of Na_2_S_2_O_8_@HTSMSEF concentration. Generally, the major type of free radical produced through Fe^2+^‐catalyzed S_2_O_8_^2−^ oxidation is •SO_4_^−^. Subsequently, •SO_4_^−^ would also catalyze the decomposition of H_2_O to produce •OH. We further designed the free radical trapping experiments using ethanol (EtOH) as •SO_4_^−^/•OH radical scavenger and tert‐butanol (TBA) as •OH scavenger. As elucidated in Figure 2G,H, the degradability of MB was decreased from ≈80% to ≈48% after adding TBA to scavenge the •OH (≈32%), whereas the degradability was ≈26% after adding EtOH to scavenge the •SO_4_^−^/•OH radicals (≈54%), implying that the Na_2_S_2_O_8_@HTSMSEF pretreated with GSH could produce both •SO_4_^−^ and •OH radicals. A similar result was also observed in the degradability of methyl orange after adding Na_2_S_2_O_8_@HTSMSEF pretreated with GSH (Figure S19). Further, the generation of •SO_4_^−^/•OH radicals in the Na_2_S_2_O_8_@HTSMSEF after GSH pretreatment was verified by electron spin resonance (ESR) spectra analysis. It was found that there were multiple peaks of •SO_4_^−^ with four sharp ones of •OH in the Na_2_S_2_O_8_@HTSMS and Na_2_S_2_O_8_@HTSMSEF pretreated with 5 mm of GSH, and the latter group exhibited the significantly stronger signals compared with the former one (Figure 2I). The result indicated the successful cycling amplification of •SO_4_^−^/•OH radicals via EG‐activated Fe^2+^‐supply‐regeneration. Once the acidity was further increased, the Na_2_S_2_O_8_@HTSMSEF successively pretreated with GSH and H_2_O_2_ presented the dramatically stronger •SO_4_^−^/•OH signals (Figure 2J), implying that the rational control of intracellular pH could maximize the Fenton catalytic efficiency while promoting the EG activation (Figure S20).

### 2.4 Intracellular Na^+^ overload and Fe^2+^ dynamics

To elucidate the superiority of Na_2_S_2_O_8_@HTSMSEF, Na_2_S_2_O_8_/Fe^2+^ and Na_2_S_2_O_8_@HTSMS‐Fe^2+^ (Na_2_S_2_O_8_@HTSMSF) were chosen as control groups. First, the cellular internalization behaviours of Na_2_S_2_O_8_@HTSMSEF were investigated by using the fluorescence probe IR820‐NHS, which labelled the amine functional groups of HTSMS. As shown in Figure S21, the nanoreactors exhibited a typical time‐dependent cellular uptake behaviour, which motivated us to study whether it could trigger intracellular Na^+^ overload and Fe^2+^ dynamics. As shown in Figure 2K,L, the Na_2_S_2_O_8_/Fe^2+^‐treated cells showed no remarkable enhancement of sodium‐binding benzofuran isophthalate (SBFI, Na^+^ indicator) and FerroOrange (Fe^2+^ indicator) fluorescence signals. By contrast, significantly stronger SBIF**/**FerroOrange fluorescence signals were observed in the Na_2_S_2_O_8_@HTSMSEF‐treated HepG2 cells. The flow cytometry detection was well aligned with the confocal observation (Figure S22). The results revealed that the Na_2_S_2_O_8_@HTSMSEF could circumvent the Na^+^, K^+^‐ATPase across the cellular membrane to enter into cells for Na^+^ overload accompanied by Fe^2+^ intake (Figure 2M).

### 2.5 In vitro anticancer activity

**FIGURE 3:**
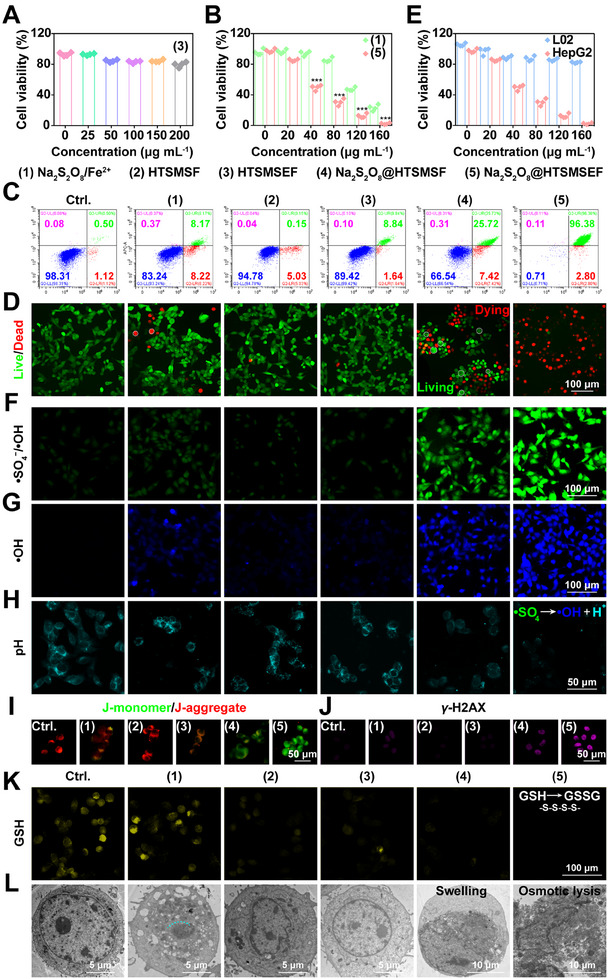
In vitro anticancer activity of Na_2_S_2_O_8_@HTSMSEF. (A) Biocompatibility of HepG2 cells incubated with HTSMSEF for 24 h. (B) Cytotoxicity of HepG2 cells treated by Na_2_S_2_O_8_/Fe^2+^ and Na_2_S_2_O_8_@HTSMSEF at various Na_2_S_2_O_8_ concentrations (*n* = 4). ***p < 0.001 (Student's *t*‐test). (C) Flow cytometry plots and (D) cell living/dying of HepG2 cells treated by different formulations. (E) Cell viability of L02 cells (human liver normal cells) and HepG2 cells treated with Na_2_S_2_O_8_/Fe^2+^ and Na_2_S_2_O_8_@HTSMSEF at various Na_2_S_2_O_8_ concentrations. (F) Confocal images of HepG2 cells treated by different formulations and stained with DCFH‐DA, indicative of intracellular •SO_4_^−^/•OH radical generation. (G) Confocal images of HepG2 cells treated by different formulations and stained with coumarin‐3‐carboxylic acid, indicative of intracellular •OH generation. (H–J) Confocal images of HepG2 cells treated by different formulations and stained with (H) BCECF‐AM, (I) JC‐1, and (J) γ‐H2AX, indicative of acidification, ΔΨm depolarization, and DNA double‐strand breakage respectively. (K) Confocal images of HepG2 cells treated by different formulations followed by staining with ThiolTracker violet, indicative of intracellular GSH‐depleting ability. (L) Bio‐TEM images of HepG2 cells treated by different formulations.

To investigate the anticancer activity of Na_2_S_2_O_8_@HTSMSEF at the cellular level, the cell biocompatibility of HTSMSEF was first conducted on HepG2 cells by CCK‐8 assay. No significant cytotoxicity was observed below 200 µg mL^‐1^ of HTSMSEF (Figure 3A). Furthermore, the cell cytotoxicity was investigated to compare Na_2_S_2_O_8_/Fe^2+^ with Na_2_S_2_O_8_@HTSMSEF (Figure 3B and Figure S23). As the Na_2_S_2_O_8_ concentration was increased to 40 µg mL^‐1^, ≈50% of HepG2 cells treated with Na_2_S_2_O_8_@HTSMSEF survived after treatment for 24 h. By contrast, the viability of cells treated with Na_2_S_2_O_8_/Fe^2+^ was higher than 90%, which was likely explained by the limited delivery ability. The result indicated the potent cancer cell‐killing ability of Na_2_S_2_O_8_@HTSMSEF, presenting a 1.9‐fold improvement compared with Na_2_S_2_O_8_/Fe^2+^ at the cellular level. In addition, both flow cytometry plots and calcein‐AM/PI assays were used to verify this finding (Figure 3C,D). Notably, it was discovered that Na_2_S_2_O_8_@HTSMSEF showed significantly lower cytotoxicity at various concentrations towards human liver normal cell line (L02 cells) compared with HepG2 cells ranging from 20 to 160 µg mL^‐1^ of Na_2_S_2_O_8_ (Figure 3E). After 24 h incubation of Na_2_S_2_O_8_@HTSMSEF at 160 µg mL^‐1^ of Na_2_S_2_O_8_ concentration, HepG2 cells were almost completely killed. On the contrary, ≈80% of L02 cells survived, indicating cancer cell‐killing specificity Na_2_S_2_O_8_@HTSMSEF. One conceivable reason was that both the intracellular anti‐oxidative ability and Na^+^ contents inside normal cells were significantly higher than those in tumour cells.^[^ ^13^, ^31^, ^32^, ^33^ ^]^ So, compared with normal cells, tumour cells were particularly vulnerable to both the oxidative stress and osmolarity increase resulting from the Na_2_S_2_O_8_@HTSMSEF treatment.

Then the therapeutic mechanism of Na_2_S_2_O_8_@HTSMSEF was further explored. So the intracellular •SO_4_^−^/•OH generation after Na_2_S_2_O_8_@HTSMSEF treatment was investigated by 2′,7′‐dichlorodihydrofluorescein diacetate (DCFH‐DA, labelling •SO_4_^−^/•OH) and coumarin‐3‐carboxylic acid (labelling only •OH^[^ ^34^, ^35^, ^36^ ^]^). Compared with Na_2_S_2_O_8_/Fe^2+^‐treated HepG2 cells, Na_2_S_2_O_8_@HTSMSF‐treated cells produced noticeable •SO_4_^−^ and •OH, and significantly higher levels were observed in the Na_2_S_2_O_8_@HTSMSEF‐treated cells (Figure 3F,G, and Figure S24). Further, we found that the pre‐treatment of vitamin C (ROS scavenging agent) significantly attenuated cell death (Figure S25). So, one of the cytotoxic mechanisms of Na_2_S_2_O_8_@HTSMSEF was derived from ROS generation. In addition, by using an intracellular pH fluorescence probe (BCECF‐AM), significant acidification was found in the Na_2_S_2_O_8_@HTSMSEF groups (Figure 3H), indicating the successful intracellular •SO_4_^−^‐catalyzed H_2_O decomposition. Moreover, the mitochondrial membrane potential (ΔΨm) depolarization (Figure 3I), ATP production inhibition (Figure S26), DNA double‐strand breakage (Figure 3J and Figure S27), GSH exhaustion (Figure 3K and Figure S28), and G2/M cell‐cycle arrest (Figure S29) after Na_2_S_2_O_8_@HTSMSEF treatment were also involved in inducing the cellular death. From the Bio‐TEM observation (Figure 3L), not only the cellular membrane was broken due to the swelling (discussed below), but also the organelles were severely damaged after Na_2_S_2_O_8_@HTSMSEF treatment. In contrast, the cells after other treatments were kept in nearly intact cellular morphology with normal organelles. Thus, the Na_2_S_2_O_8_@HTSMSEF nanoreactors resulted in strong damage to cancer cells.

### 2.6 Evoking pyroptosis and mechanistic investigation

**FIGURE 4:**
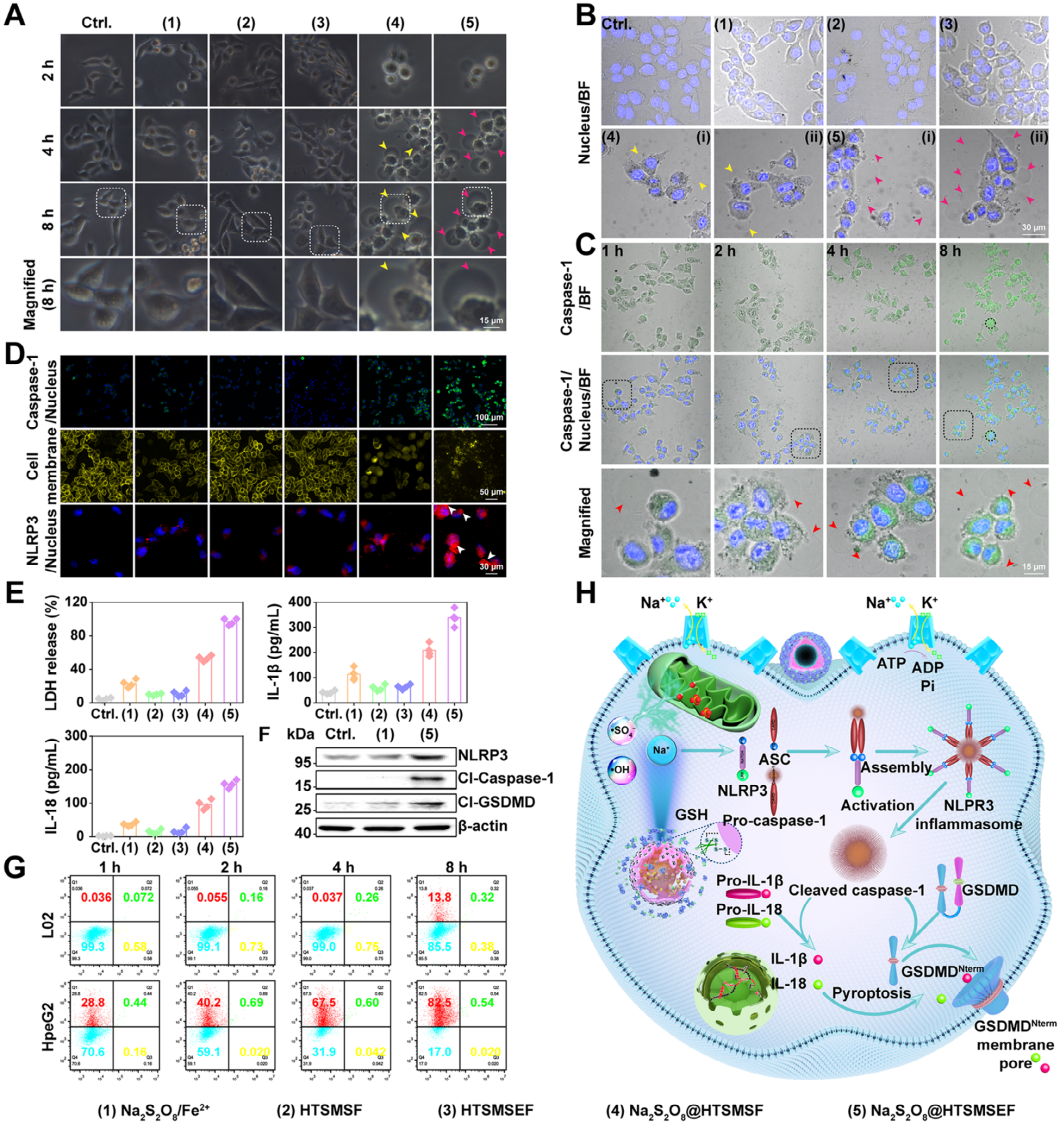
Pyroptosis evoked by Na_2_S_2_O_8_@HTSMSEF. (A) Dynamic optical imaging of pyroptosis in HepG2 cells treated by different formulations including (1) Na_2_S_2_O_8_/Fe^2+^, (2) HTSMSF, (3) HTSMSEF, (4) Na_2_S_2_O_8_@HTSMSF, and (5) Na_2_S_2_O_8_@HTSMSEF. The arrows indicate the ballooning bubbles. (B) Confocal observation of pyroptosis in HepG2 cells treated by different formulations. (C) Time‐lapsed confocal observation of caspase‐1 expression in HepG2 cells after treatment of Na_2_S_2_O_8_@HTSMSEF using immunofluorescence. (D) Caspase‐1 cleavage, cell membrane integrity, and NLRP3 inflammasome activation of HepG2 cells after different treatments. The arrows indicate speck‐like aggregates of NLRP3. (E) LDH release and IL‐1β/IL‐18 leakage of HepG2 cells after different treatments using an ELISA assay kit (*n* = 3). *p < 0.05 (Student's *t*‐test). (F) Western blot assay of the pyroptosis signalling pathway (NLRP3 inflammasome activation, caspase‐1 cleavage, and GSDMD‐N release) in HepG2 cells treated by different formulations. (G) Flow cytometry plots of L02 and HepG2 cells treated by Na_2_S_2_O_8_@HTSMSEF. (H) Illustration of activation of pyroptosis pathway by Na_2_S_2_O_8_@HTSMSEF.

Very recently, it has been proven that an osmolarity surge could activate the NLRP3 inflammasome assembly to induce pyroptosis,^[^ ^13^, ^37^, ^38^ ^]^ which is characterized by the swelling of cells with the formation of giant bubbles.^[^ ^39^, ^40^, ^41^ ^]^ HepG2 cells were incubated with different formulations at a relatively high Na_2_S_2_O_8_ concentration (80 µg mL^−1^) to monitor the cellular morphologic change. Noteworthy, by direct optical observation, it was found that Na_2_S_2_O_8_@HTSMSF and Na_2_S_2_O_8_@HTSMSEF‐treated HepG2 cells swelled into a nearly spherical cell morphotype accompanied by the formation of transparent large ballooning bubbles on the membrane surface (Figure 4A). The result implied that Na_2_S_2_O_8_@HTSMSEF could efficiently induce pyroptosis. Confocal observation via bright‐field imaging with caspase‐1 staining (discussed below) further proved the results (Figure 4B,C, and Figure S30). To investigate the cellular membrane integrity, HepG2 cells were stained with rhodamine‐WGA (plasma membrane probe). It was found that the Na_2_S_2_O_8_@HTSMSEF treatment induced severe cell membrane damage as evidenced by the weakest and messiest red fluorescence signals (Figure 4D and Figure S31–S33).

Gasdermin‐D (GSDMD), identified in 2015, is a pyroptosis execution protein (key “killer protein” to execute the pyroptosis) cleaved by “NLRP3 inflammasome‐activated caspase‐1”.^[^ ^41^, ^42^, ^43^ ^]^ During pyroptosis, the cleaved GSDMD at the N‐terminus (denoted as GSDMD‐N) could transfer to the membrane phospholipid to create the transmembrane pores,^[^ ^40^, ^44^ ^]^ accompanied by the secretion and release of lactate dehydrogenase (LDH) from the cells. By using an LDH release assay kit, we found that the LDH release in Na_2_S_2_O_8_@HTSMSEF‐treated HepG2 cells was 1.83‐fold and 4.18‐fold higher than that in Na_2_S_2_O_8_/Fe^2+^ and Na_2_S_2_O_8_@HTSMSF‐treated cells, respectively (Figure 4E). Moreover, the pre‐treatment of Ac‐YVAD‐CMK (caspase‐1 inhibitor)^[^ ^45^ ^]^ significantly alleviated the release of LDH outside the cellular membrane (Figure S34). To further reveal the underlying molecular mechanism behind pyroptosis, we performed western‐blotting analysis to determine the expression of related key proteins.^[^ ^40^, ^41^, ^42^, ^43^, ^44^ ^]^ ‐terminal domain of GSDMD compared with the Na_2_S_2_O_8_/Fe^2+^ group (Figure 4F). Moreover, the immunocytochemical staining proved that the cells treated with Na_2_S_2_O_8_@HTSMSEF potently elevated the activity of both NLRP3 inflammasome (with speck‐like aggregates) and caspase‐1 compared with the other groups (Figure 4D). The result indicated that Na_2_S_2_O_8_@HTSMSEF activated the assembly of NLRP3 inflammasome and subsequently activated the caspase‐1 to drive the pyroptosis of cancer cells. Furthermore, the expression levels of cytosolic pro‐inflammatory cytokines (IL‐1β and IL‐18) in Na_2_S_2_O_8_@HTSMSEF‐treated cells were significantly higher than those in the other groups (Figure 4E), implying the Na_2_S_2_O_8_@HTSMSEF remarkably increased the IL‐1β/IL‐18 leakage. Additionally, through the flow cytometry detection, we found a majority of HepG2 cells after treatment of Na_2_S_2_O_8_@HTSMSEF at 120 µg mL^−1^ of Na_2_S_2_O_8_ concentration were in the pyroptosis (programmed necrosis) state (Figure 4G). These results proved that the Na_2_S_2_O_8_@HTSMSEF could efficiently induce the pyroptosis via activating the NLRP3 inflammasome assembly, then triggering the caspase‐1 activation, and lastly driving the N‐terminal GSDMD cleavage to release the IL‐1β/IL‐18 outside of the cellular membrane (Figure 4H).

### 2.7 In vivo tumour accumulation, pharmacokinetics, and bio‐distribution

**FIGURE 5:**
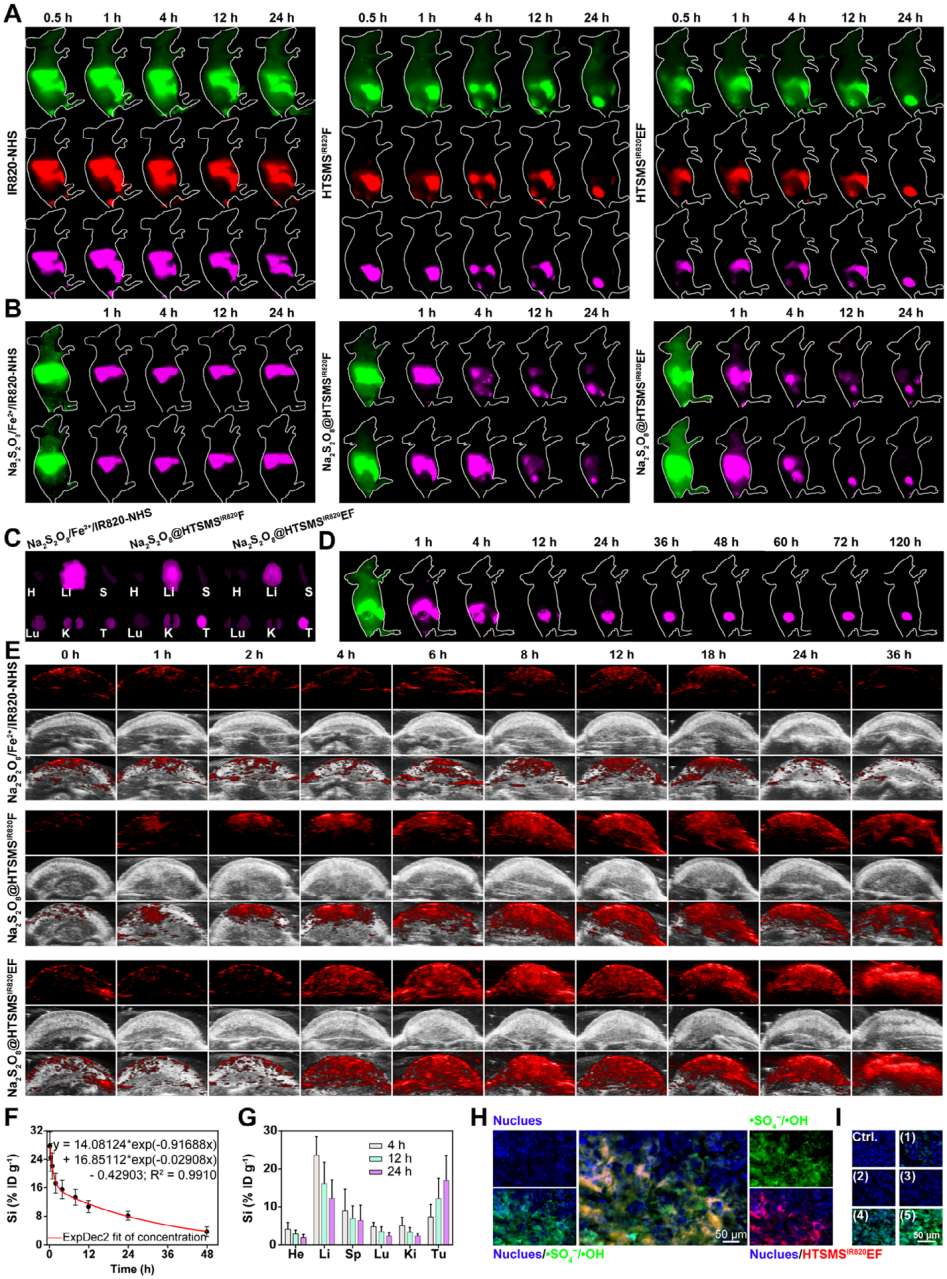
In vivo tumour accumulation and bio‐distribution of Na_2_S_2_O_8_@HTSMSEF. (A) Dynamic in vivo NIR‐II fluorescence imaging of mice intravenously injected with HTSMS^IR820^EF (*n* = 3). IR820‐NHS and HTSMS^IR820^F were utilized as controls. The excitation wavelength was 808 nm, and the central wavelength of the long wave pass filter was 880 nm (false green), 1000 nm (false red), and 1250 nm (false purple). (B) Dynamic in vivo NIR‐II fluorescence imaging of mice intravenously injected with Na_2_S_2_O_8_@HTSMS^IR820^EF (*n* = 2). Na_2_S_2_O_8_/Fe^2+^/IR820‐NHS and Na_2_S_2_O_8_@HTSMS^IR820^F were used as controls. (C) Ex vivo NIR‐II fluorescence images of major tissues and tumour dissected from mice intravenously injected with Na_2_S_2_O_8_@HTSMS^IR820^EF at 24 h post‐injection. Na_2_S_2_O_8_/Fe^2+^/IR820‐NHS and Na_2_S_2_O_8_@HTSMS^IR820^F were used as controls. (D) Long‐term time‐lapsed NIR‐II fluorescence imaging of mice intravenously injected with Na_2_S_2_O_8_@HTSMS^IR820^EF. (E) Dynamic photoacoustic imaging of tumour region of mice injected with Na_2_S_2_O_8_@HTSMS^IR820^EF. (F) In vivo pharmacokinetics and (G) bio‐distribution of mice intravenously injected with Na_2_S_2_O_8_@HTSMS^IR820^EF at different time intervals based on ICP‐OES‐determined element levels (*n* = 3). *p < 0.05 (Student's *t*‐test). (H, I) ROS staining of tumour slices from mice intravenously injected with (H) Na_2_S_2_O_8_@HTSMS^IR820^EF and (I) different formulations: (1) Na_2_S_2_O_8_/Fe^2+^, (2) HTSMSF, (3) HTSMSEF, (4) Na_2_S_2_O_8_@HTSMSF, (5) and Na_2_S_2_O_8_@HTSMSEF.

Before performing the antitumor effect in vivo, we estimate the hemocompatibility of Na_2_S_2_O_8_@HTSMSEF by hemolysis assay. It was found that the Na_2_S_2_O_8_@HTSMSEF displayed no significant damage to red cell membranes even at the concentration of 400 µg mL^−1^ (Figure S35). We further investigated tumour accumulation of Na_2_S_2_O_8_@HTSMS^IR820^EF after intravenous administration into HepG2 tumour‐bearing nude mice by NIR‐II fluorescence imaging. Compared with the IR820‐NHS‐treated mice, the HTSMS^IR820^F and HTSMS^IR820^EF‐treated mice showed significantly stronger NIR‐II fluorescence distributed at the tumour sites (Figure 5A and Figure S36), which could be explained by the enhanced penetration and retention (EPR) effect of nano‐sized HTSMS.^[^ ^46^, ^47^ ^]^ A similar result was also observed in the Na_2_S_2_O_8_@HTSMS^IR820^F and Na_2_S_2_O_8_@HTSMS^IR820^EF‐treated mice in comparison with Na_2_S_2_O_8_/Fe^2+^/IR820‐NHS‐treated mice (Figure 5B,C). Notably, we performed the long‐term time‐lapsed NIR‐II fluorescence imaging and found the accumulation and retention of Na_2_S_2_O_8_@HTSMS^IR820^EF in the tumour regions could be continued for 120 h (Figure 5D). The result could be explained by the effect of “polyphenol” lation or “hydroxy” lation, which was remarkably similar to PEGylation and could also prolong the blood circulation life of nanocarriers.^[^ ^48^, ^49^, ^50^, ^51^, ^52^, ^53^ ^]^ In addition, the photoacoustic imaging results indicated a stark accumulation enhancement of Na_2_S_2_O_8_@HTSMS^IR820^F and Na_2_S_2_O_8_@HTSMS^IR820^EF in the tumour areas in comparison with Na_2_S_2_O_8_/Fe^2+^/IR820‐NHS group (Figure 5E). To investigate the relationship between the imaging signal and distribution, the circulation lifetime (T_1/2_) and bio‐distribution of Na_2_S_2_O_8_@HTSMSEF were explored. Through a double‐compartment pharmacokinetic model, T_1/2_ was calculated to be 3.66 h (Figure 5F). Besides, the results regarding element levels (Figure 5G) and ROS levels (Figure 5H,I) by ICP‐OES analysis and confocal observation coincided with the above imaging results, proving the superior accumulation efficiency of Na_2_S_2_O_8_@HTSMSEF within the tumours.

### 2.8 In vivo antitumor efficacy and safety

**FIGURE 6:**
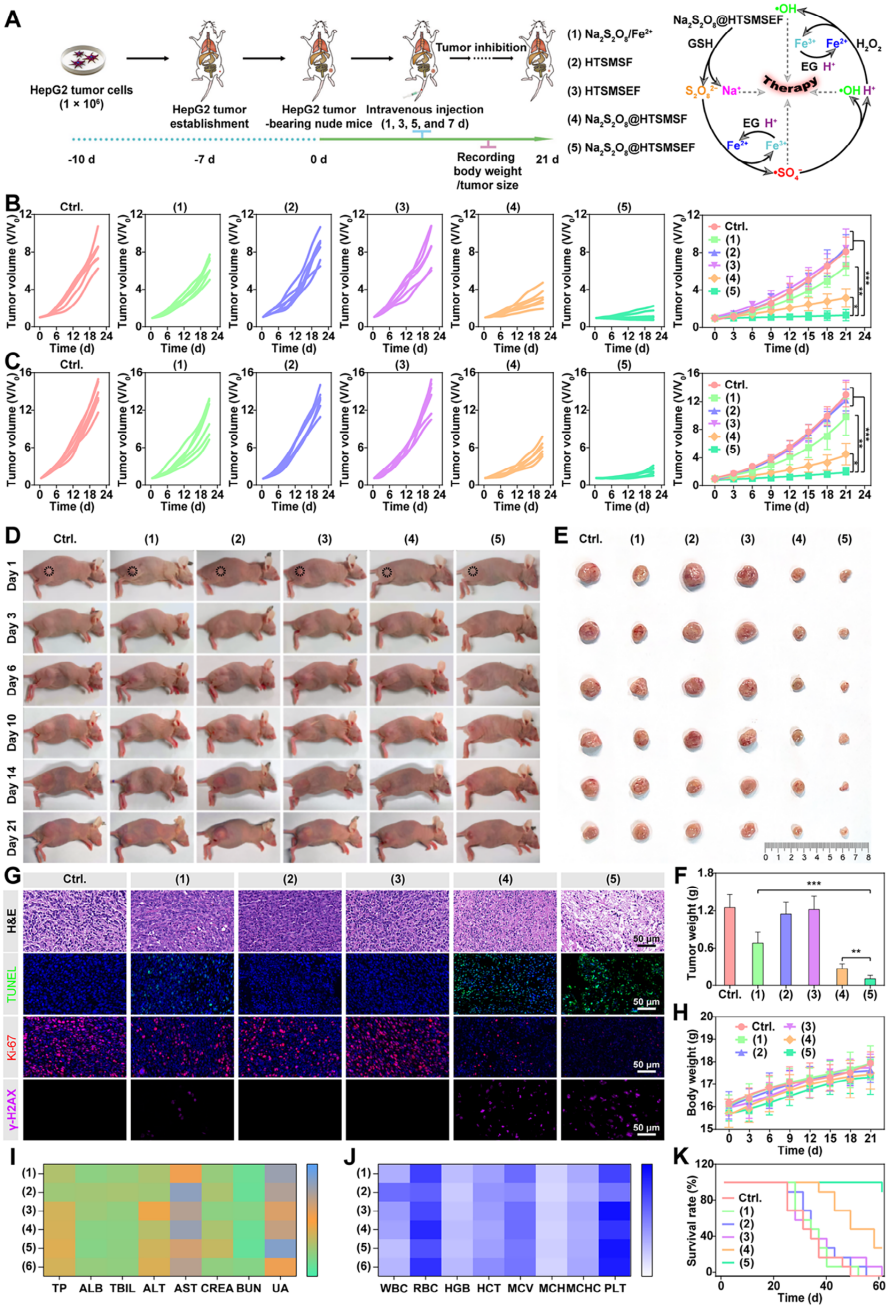
In vivo tumour accumulation and therapeutic efficiency of Na_2_S_2_O_8_@HTSMSEF. (A) Illustration of therapeutic schedule and mechanism. (B,C) tumour volume curves (B: HepG2 tumour; C: 4T1 tumour), (D) total‐body photographs, (E) dissected tumour photographs, and (F) dissected tumour weight of mice after different treatments during 21‐day therapy (*n* = 6). (G) H&E/TUNEL/Ki‐67 and γ‐H2AX staining in tumour slices from mice after different treatments. (H) Body weight variations of mice after different treatments during 21‐day therapy (*n* = 6). (I) Hematological and (J) blood biochemical indexes of mice after different treatments. (K) Survival percentages of mice after different treatments during 60‐day therapy (*n* = 6). *p < 0.05, **p < 0.01, and ***p < 0.001 (Student's *t*‐test).

The robust anticancer activity in vitro of Na_2_S_2_O_8_@HTSMSEF motived us to explore its antitumor effect in vivo. The therapeutic schedule was developed as described in Figure 6A. HepG2 liver tumour‐bearing nude mouse model was divided into six groups followed by the injection with 0.9% NaCl, Na_2_S_2_O_8_/Fe^2+^, HTSMSF, HTSMSEF, Na_2_S_2_O_8_@HTSMSF, and Na_2_S_2_O_8_@HTSMSEF at the equivalent dosage of Na_2_S_2_O_8_ or HTSMS. As presented in Figure 6B, the tumour growth was very rapid in the 0.9% NaCl group. The treatments of HTSMS and HTSMSEF presented slight control on tumour growth, implying their weak antitumor efficacy. On the contrary, the Na_2_S_2_O_8_/Fe^2+^, Na_2_S_2_O_8_@HTSMSF, and Na_2_S_2_O_8_@HTSMSEF treatments effectively suppressed tumour growth. Notably, the average HepG2 tumour volume in the Na_2_S_2_O_8_@HTSMSEF‐treated mice on day 21 was 41.7% and 20.4% of that in the Na_2_S_2_O_8_@HTSMSF and Na_2_S_2_O_8_/Fe^2+^ groups, respectively. A similar phenomenon was also shown in the 4T1 breast tumour‐bearing nude mouse model (Figure 6C), validating the universality of the Na_2_S_2_O_8_@HTSMSEF treatment strategy. Moreover, both the total‐body and dissected tumour photographs intuitively displayed the superior tumour growth inhibition of Na_2_S_2_O_8_@HTSMSEF treatment in compared to the other treatments (Figure 6D–F). Furthermore, the results of hematoxylin and eosin (H&E)/TUNEL/Ki‐67 and γ‐H2AX staining of tumour slices proved that the Na_2_S_2_O_8_@HTSMSEF produced the most cellular damage via pyroptosis/apoptosis (Figure 6G). These outcomes implied that the Na_2_S_2_O_8_@HTSMSEF multiply amplified the dynamic therapeutic efficiency via cascade‐responsive dual cycling amplification of •SO_4_^−^/•OH accompanied by osmolarity and redox homeostasis dysregulation.

During the treatment, the Na_2_S_2_O_8_@HTSMSEF injection did not cause a significant decrease in body weight (Figure 6H), which was consisted with some reported studies.^[^ ^54^, ^55^, ^56^ ^]^ Moreover, the H&E staining of major tissues in Na_2_S_2_O_8_@HTSMSEF‐treated HepG2 tumour‐bearing mice revealed no apparent damage to the major tissues (Figure S37). In addition, through the blood cell counts and systemic cytokine ELISA assay, the Na_2_S_2_O_8_@HTSMSEF treatment did not induce visible systemic inflammation/toxicity (Figure 6I,J). Furthermore, the Na_2_S_2_O_8_@HTSMSEF injection extended the median survival time of mice from 21 days to 58.5 days compared with the 0.9%NaCl treatment, while Na_2_S_2_O_8_@HTSMSF and Na_2_S_2_O_8_/Fe^2+^ treatment respectively prolonged the median survival time to 45 and 27 days (Figure 6K). The results revealed that the Na_2_S_2_O_8_@HTSMSEF possessed a convincing biosafety profile.

## 3 CONCLUSIONS

In summary, we intelligently develop a tumour‐microenvironment‐driven yolk‐shell nanoreactor that could realize the cascade‐responsive dual cycling amplification of •SO_4_^−^/•OH to achieve a new type of tumour‐dynamic therapy. With the cavity‐confined effect, the nanoreactor is first constructed by encapsulating Na_2_S_2_O_8_ nanocrystals into hollow tetra‐sulphide‐introduced mesoporous organosilica, and then it is enclosed by EG‐Fe(II). The nanoreactor can be systemically delivered to tumour sites to specifically release Na^+^/S_2_O_8_^2−^/Fe^2+^ and EG upon the intracellular GSH triggering. Thus, a sequence of cascade reactions will be activated to generate and circularly amplify •SO_4_^−^/•OH radicals via Fe^2+^‐catalyzed S_2_O_8_^2−^ oxidation, •SO_4_^−^‐catalyzed H_2_O decomposition, proton‐intensified Fenton oxidation, and EG‐activated Fe^2+^‐supply‐regeneration. Meanwhile, the overloaded Na^+^ and tetra‐sulphide bridge can respectively cause the osmolarity imbalance and GSH depletion to further amplify oxidative stress. In vitro/in vivo results verify that the nanoreactor efficiently inhibits tumour growth by multiple amplified oxidative stress, which will provide a new strategy for high‐efficiency persistent synergistic dynamic therapy.

## 4 EXPERIMENTAL SECTION

### 4.1 Materials

Cetyltrimethylammonium bromide (CTAB), triethanolamine (TEA, 99.5%), 3‐aminopropyltriethoxysilane (APTES), bis[3‐(triethoxysilyl)propyl] tetra‐sulphide (BTES), tetraethyl orthosilicate (TEOS), epigallocatechin gallate (EG), and ferrous chloride were purchased from Sigma‐Aldrich Trading Co., Ltd. (Shanghai, China). Sodium palmitate (C_15_H_31_COONa), ammonium peroxodisulfate ((NH_4_)_2_S_2_O_8_), lecithin, and oleylamine were obtained from Aladdin Reagent Co., Ltd. (Shanghai, China). FerroOrange and rhodamine‐WGA were acquired from Maokang Biotechnology Co., Ltd (Shanghai, China). SBFI, Thiol Tracker Violet, SYTOX green, SOSG, and Molecular Probes ATP determination assay kit were acquired from Thermo Fisher Scientific Co., Ltd. (Shanghai, China). DCFH‐DA, BCECF‐AM, JC‐1, dihydroethidium, DNA damage assay kit, CCK‐8 cell viability assay kit, calcein‐AM/propidium iodide (PI) assay kit, and Annexin V‐FITC/PI apoptosis assay kit were acquired from Beyotime Biotechnology Co., Ltd (Shanghai, China). Coumarin‐3‐carboxylic acid was purchased from MedChem Express Co., Ltd (Shanghai, China). FAM‐FLICA™ caspase‐1 assay kit was obtained from Huicheng Biotechnology Co., Ltd (Shanghai, China).

### 4.2 Preparation of SS@TSMS

First, the SS was synthesized via a modified Stöber approach.^[^ ^24^ ^]^ Then, the SS@TSMS was synthesized by using a modified biphase stratification method.^[^ ^57^ ^]^ 20 mg of SS was added into 10 mL of water with the addition of 150 mg of CTAB and 20 µL of TEA at 60°C. Afterward, the mixture of 0.9 mL of BTES/TEOS (1: 1, v/v) and 3 mL of cyclohexane was added while stirring for 24 h. Lastly, the obtained SS@TSMS was washed using ethanol by centrifugation and stored at 4°C.

### 4.3 Preparation of HTSMS

40 mg of SS@TSMS was added into 4 mL of sodium hydroxide (0.1 m) while stirring at 60°C for 4 h. Subsequently, the obtained HTSMS was washed with ethanol by centrifugation and stored at 4°C. In addition, 10 mg of HTSMS nanoparticles and 100 µL of APTES were added into 10 mL of ethanol. After vigorous stirring at 70°C for 24 h, the obtained amine‐functionalized HTSMS nanoparticles were washed with ethanol by centrifugation for subsequent fluorescence labelling.

### 4.4 Synthesis of Na_2_S_2_O_8_@HTSMS

The Na_2_S_2_O_8_@HTSMS nanoparticles were prepared via a cavity‐confined in situ chemical synthesis approach. 30 mg of HTSMS was dispersed in 13 mL of *n*‐hexane/ethanol/oleylamine (10: 2: 1, v/v/v) via ultrasonication for 10 min with the addition of 30 mg of C_15_H_31_COONa. Then, 23 mg of (NH_4_)_2_S_2_O_8_ and 10 mg of lecithin were added and kept stirring for 12 h. Afterward, the obtained Na_2_S_2_O_8_@HTSMS nanoparticles were washed with *n*‐hexane/ethanol by centrifugation and stored at 4°C.

### 4.5 Fabrication of Na_2_S_2_O_8_@HTSMSEF

The Na_2_S_2_O_8_@HTSMSEF nanoreactors were prepared via an interface cross‐linking approach. 50 mg of Na_2_S_2_O_8_@HTSMSF was dispersed in 12 mL of *n*‐hexane/ethanol (10: 2, v/v) via ultrasonication for 30 min with the addition of 20 mg of polyphenol‐structured EG for surface adhesion. Then, 10 mg of ferrous chloride was added and kept stirring for 6 h. Afterward, the obtained Na_2_S_2_O_8_@HTSMSEF nanoreactors were washed with *n*‐hexane/ethanol by centrifugation.

### 4.6 Characterization

Transmission electron microscopy (TEM) images, selected area electron diffraction (SAED) patterns, and energy‐dispersive X‐ray (EDX) element mappings were carried out on a Talos F200 field emission transmission electron microscopy (US). Dynamic light scattering (DLS) was tested by using a Malvern Zetasizer Nano ZS (UK). N_2_ adsorption‐desorption isotherm was tested on a Tristar 3000 system (US). UV–vis spectra were tested on an Agilent Cary 5000 spectrophotometer (US). X‐ray diffraction (XRD) spectra were recorded by a Bruker AXS‐D8 Advance diffractometer with Cu‐Kα source (Germany). X‐ray photoelectron spectra (XPS) were obtained on Thermo Scientific K‐Alpha+ with Al‐Kα source (US). Electron spin resonance (ESR) spectra were acquired using a Bruker EMX‐10/12 spectrometer (Germany). Confocal microscopy imaging was carried out on a Nikon A1RMP multiphoton confocal microscopy system (Japan). Inductively coupled plasma optical emission spectrum (ICP‐OES) was determined by an Agilent 5100 inductively coupled plasma optical emission spectrometer (Switzerland). NIR‐II fluorescence imaging was performed on a NIR‐OPTICS Series III 900/1700 (China). Photoacoustic imaging was performed on a VisualSonics Vevo‐2100 imaging system (Canada).

### 4.7 GSH‐responsive degradation/Na^+^ release and GSH exhaustion

The Na_2_S_2_O_8_@HTSMSEF nanoreactors were added into ammonium acetate buffer containing various GSH concentrations and stirred for different times. Next, the supernatant was collected by centrifugation and the Na^+^ concentration was determined by the Na^+^ electrode. In addition, the Na_2_S_2_O_8_@HTSMSEF was added into 10 mM of GSH followed by the addition of 0.2 mm of DTNB to test the sulphydryl moiety of GSH. The absorbance at 412 nm was determined by a UV–vis spectrophotometer.

### 4.8 Cell culture

The human liver cancer cell line (HepG2 cells), human liver normal cell line (L02 cells), and mouse breast cancer cell line (4T1 cells) were provided from the cell bank of the Chinese Academy of Sciences.

### 4.9 Intracellular Na^+^/Fe^2+^ levels

The cells were seeded in six‐well plates. Next, the cells were treated by Na_2_S_2_O_8_@HTSMSEF for 8 h and then incubated with SBFI (Na^+^ indicator probe) and FerroOrange (Fe^2+^ indicator probe) for 0.5 h, respectively. Finally, the cells were washed by PBS for confocal observation and subsequently treated with trypsin for flow cytometry detection.

### 4.10 In vitro cytotoxicity

The cells were seeded in a 96‐well plate. Then, the cells were treated by Na_2_S_2_O_8_@HTSMSEF at different concentrations for another 24 h. After discarding the culture medium, the CCK‐8 assay was assessed according to the instruction and operation manual.

### 4.11 In vitro apoptosis analysis and living/dying observation

The cells were seeded in six‐well plates. Then, the cells were treated by 0.9% NaCl, Na_2_S_2_O_8_/Fe^2+^, HTSMSF, HTSMSEF, Na_2_S_2_O_8_@HTSMSF, and Na_2_S_2_O_8_@HTSMSEF for another 8 h. For apoptosis analysis, the cells were collected using trypsin, washed by PBS, resuspended in Annexin‐binding buffer, stained with Annexin V‐FITC/PI according to the instruction and operation manual, and analyzed by flow cytometry. For living/dying observation, the cells were washed with PBS, stained with calcein‐AM/PI according to the instruction and operation manual, and observed by confocal microscopy.

### 4.12 Intracellular ROS generation and ΔΨm change

The cells were seeded in six‐well plates. Afterward, the cells were treated by 0.9% NaCl, Na_2_S_2_O_8_/Fe^2+^, HTSMSF, HTSMSEF, Na_2_S_2_O_8_@HTSMSF, and Na_2_S_2_O_8_@HTSMSEF for 8 h. After discarding the medium, the DCFH‐DA probes were added and incubated for 20 min for detection of intracellular ROS by using confocal microscopy and flow cytometer. In addition, the JC‐1 probe was added for 0.5 h incubation to detect the ΔΨm change by using confocal microscopy.

### 4.13 Intracellular GSH exhaustion

The cells were seeded in six‐well plates. Next, the cells were treated by 0.9% NaCl, Na_2_S_2_O_8_/Fe^2+^, HTSMSF, HTSMSEF, Na_2_S_2_O_8_@HTSMSF, and Na_2_S_2_O_8_@HTSMSEF for another 8 h. After discarding the medium, the ThiolTracker violet probe was added for 0.5 h incubation. Lastly, the cells were washed by PBS for confocal observation.

### 4.14 Intracellular DNA damage and ATP level

The cells were seeded in 6‐well plates. After 24 h of culture, the cells were treated with 0.9% NaCl, Na_2_S_2_O_8_/Fe^2+^, HTSMSF, HTSMSEF, Na_2_S_2_O_8_@HTSMSF, and Na_2_S_2_O_8_@HTSMSEF for 8 h. Subsequently, the intracellular DNA double‐strand breakage and ATP level were respectively investigated by using DNA damage assay kit (by γ‐H2AX Immunofluorescence) and Molecular Probes ATP determination assay kit according to the instruction and operation manual. Lastly, the cells were washed by PBS for confocal microscopy observation and flow cytometer analysis.

### 4.15 Intracellular LDH release, caspase‐1 activation, and IL‐1β secretion

The cells were seeded in 6‐well plates. Afterward, the cells were treated by 0.9% NaCl, Na_2_S_2_O_8_/Fe^2+^, HTSMSF, HTSMSEF, Na_2_S_2_O_8_@HTSMSF, and Na_2_S_2_O_8_@HTSMSEF for 8 h. Lastly, the LDH release, caspase‐1 activation, and IL‐1β secretion were detected using LDH release assay kit, enzyme‐linked immunosorbent assay (ELISA) assay kit, and FAM‐FLICA caspase‐1 assay kit according to the instruction and operation manual.

### 4.16 Animal model establishment

The animal experiment protocols were approved by the Animal Ethics Committee of the Mengchao Hepatobiliary Hospital of Fujian Medical University. The female BALB/c nude mice (aged 4–6 weeks) were purchased from Guangzhou Yaokang Biotechnology Co., Ltd. (Guangzhou, China). The cells were subcutaneously injected into the side of mice. The tumour size was recorded by a digital calliper, and the tumour volume was calculated using the formula: volume = length × width^2^/2.

### 4.17 In vivo pharmacokinetics and bio‐distribution

The tumour‐bearing female BALB/c nude mice (aged 6–8 weeks) were intravenously injected by 200 µL of Na_2_S_2_O_8_@HTSMSEF, and the blood was taken to be diluted to 1 mL with PBS at different time points. Afterward, the silicon concentration was determined by ICP‐OES. Additionally, the major tissues and tumours were dissected at different time intervals, homogenized, and digested for ICP‐OES analysis.

### 4.18 In vivo imaging

The tumour‐bearing female BALB/c nude mice (aged 6–8 weeks) were intravenously injected by 200 µL of Na_2_S_2_O_8_@HTSMS^IR820^EF (IR820‐NHS was utilized to label the amine functional groups of HTSMS). For NIR‐II fluorescence imaging, the whole body were in vivo imaged at different time intervals on NIR‐OPTICS Series III 900/1700 Imaging System (excitation wavelength: 808 nm; software: PSViewer), and the major tissues and tumours were dissected followed by ex vivo fluorescence imaging after 24 h. For photoacoustic imaging, the tumour area was imaged at different time intervals by using the FUJIFILM VisualSonics Vevo 2100 Imaging System (excitation wavelength: 800 nm; software: Vevo LAB).

### 4.19 In vivo anticancer effects

The tumour‐bearing female BALB/c nude mice (aged 6–8 weeks) were stochastically divided into six groups (*n* = 6) followed by the intravenous administration with 200 µL of 0.9% NaCl, Na_2_S_2_O_8_/Fe^2+^, HTSMS, HTSMS‐EG/Fe(II), Na_2_S_2_O_8_@HTSMSF, and Na_2_S_2_O_8_@HTSMSEF at the equivalent HTSMS or Na_2_S_2_O_8_ dosage. The tumour sizes and body weights of different groups were monitored every three days, and euthanasia was performed on day 21. Then the tumour tissues were collected to perform the hematoxylin and eosin (H&E)/TUNEL/Ki‐67 and γ‐H2AX assays to prove the anticancer effects.

### 4.20 In vivo biosafety

The healthy female BALB/c nude mice (aged 6–8 weeks) were administrated with Na_2_S_2_O_8_@HTSMSEF by intravenous injection. The blood and major tissues were respectively collected for haematology analysis (detecting the haematological/serum biochemical parameters) and histological examination (detecting the pathological change by H&E assays).

### 4.21 Statistical analysis

Quantitative data were indicated as mean ± standard deviation (SD). Statistical significance was assessed by the Student's *t*‐test and assumed at a value of *p < 0.05, ** *p* < 0.01, ***p < 0.001.

## CONFLICT OF INTEREST STATEMENT

The authors declare no conflicts of interest.

## ETHICS STATEMENT

All animal experiments have been approved (Approved No. 2020125) by the Animal Ethics Committee of the Mengchao Hepatobiliary Hospital of Fujian Medical University.

## Supporting information

Supporting Information

## Data Availability

All data required to reproduce these findings are included in the article and the Supporting Information

## References

[1] B. Liu , S. Liang , Z. Wang , Q. Sun , F. He , S. Gai , P. Yang , Z. Cheng , J. Lin , Adv. Mater. 2021, 33, 2101223. 10.1002/adma.202101223 34145652

[2] W. Sun , C. Chu , S. Li , X. Ma , P. Liu , S. Chen , H. Chen , Adv. Drug Delivery Rev. 2022, 192, 114643. 10.1016/j.addr.2022.114643 36493905

[3] Q. Yan , C. Lian , K. Huang , L. Liang , H. Yu , P. Yin , J. Zhang , M. Xing , Angew. Chem., Int. Ed. 2021, 60, 17155. 10.1002/anie.202105736 34041830

[4] Z. M. Tang , Y. Y. Liu , D. L. Ni , J. J. Zhou , M. Zhang , P. R. Zhao , B. Lv , H. Wang , D. Y. Jin , W. B. Bu , Adv. Mater. 2020, 32, 1904011. 10.1002/adma.201904011 31793717

[5] Y. Liu , W. Zhen , Y. Wang , S. Song , H. Zhang , J. Am. Chem. Soc. 2020, 142, 21751. 33337859 10.1021/jacs.0c09482

[6] P. Avetta , A. Pensato , M. Minella , M. Malandrino , V. Maurino , C. Minero , K. Hanna , D. Vione , Environ. Sci. Technol. 2015, 49, 1043. 25535799 10.1021/es503741d

[7] C. Luo , J. Ma , J. Jiang , Y. Liu , Y. Song , Y. Yang , Y. Guan , D. Wu , Water Res. 2015, 80, 99. 25996757 10.1016/j.watres.2015.05.019

[8] J. Wang , B. Hasaer , M. Yang , R. Liu , C. Hu , H. Liu , J. Qu , Sci. Total. Environ. 2020, 713, 136530. 31951845 10.1016/j.scitotenv.2020.136530

[9] Q. Wang , W. Zhang , Z. Yang , Q. Xu , P. Yang , D. Wang , Water Res. 2018, 146, 19. 30218907 10.1016/j.watres.2018.09.015

[10] H. Liu , T. A. Bruton , F. M. Doyle , D. L. Sedlak , Environ. Sci. Technol. 2014, 48, 10330. 25133603 10.1021/es502056d PMC4151705

[11] K. H. Wei , J. Ma , B. D. Xi , M. D. Yu , J. Cui , B. L. Chen , Y. Li , Q. B. Gu , X. S. He , J. Hazard. Mater. 2022, 432, 128738. 35338938 10.1016/j.jhazmat.2022.128738

[12] Y. Liu , M. Zhang , W. Bu , VIEW 2020, 1, 18.

[13] W. Jiang , L. Yin , H. Chen , A. V. Paschall , L. Zhang , W. Fu , W. Zhang , T. Todd , K. S. Yu , S. Zhou , Z. Zhen , M. Butler , L. Yao , F. Zhang , Y. Shen , Z. Li , A. Yin , H. Yin , X. Wang , F. Y. Avci , X. Yu , J. Xie , Adv. Mater. 2019, 31, 1904058. 10.1002/adma.201904058 PMC6886716 31553099

[14] Y. Li , J. Lin , P. Wang , F. Zhu , M. Wu , Q. Luo , Y. Zhang , X. Liu , ACS Nano 2022, 16, 7380. 35435672 10.1021/acsnano.1c09496

[15] C. Ma , Y. Zhang , Y. Zhang , S. F. A. Rizvi , G. Fu , X. Liu , H. Zhang , Nanomedicine 2022, 39, 102460. 34530164 10.1016/j.nano.2021.102460

[16] A. R. Bassindale , M. Pourny , P. G. Taylor , M. B. Hursthouse , M. E. Light , Angew. Chem., Int. Ed. 2003, 42, 3488. 10.1002/anie.200351249 12900960

[17] B. Wu , J. Liang , J. Yang , C. Jia , X. J. Yang , H. Zhang , N. Tang , C. Janiak , Chem. Commun. 2008, 2008, 1762. 10.1039/b719019k 18379685

[18] A. Ren , K. R. Rajashankar , D. J. Patel , Nature 2012, 486, 85. 22678284 10.1038/nature11152 PMC3744881

[19] F. C. Meldrum , C. O'Shaughnessy , Adv. Mater. 2020, 32, 2001068. 10.1002/adma.202001068 32583495

[20] H. Wang , J. K. Keum , A. Hiltner , E. Baer , B. Freeman , A. Rozanski , A. Galeski , Science 2009, 323, 757. 19197057 10.1126/science.1164601

[21] X. Li , M. H. Yeh , Z. H. Lin , H. Guo , P. K. Yang , J. Wang , S. Wang , R. Yu , T. Zhang , Z. L. Wang , ACS Nano 2015, 9, 11056. 26469374 10.1021/acsnano.5b04486

[22] T. He , Y. Yuan , C. Jiang , N. T. Blum , J. He , P. Huang , J. Lin , Angew. Chem., Int. Ed. 2021, 60, 6047. 10.1002/anie.202015379 33295682

[23] W. Xuan , R. Pan , Y. Wei , Y. Cao , H. Li , F. S. Liang , K. J. Liu , W. Wang , Bioconjug. Chem. 2016, 27, 302. 26284503 10.1021/acs.bioconjchem.5b00259

[24] X. Li , L. Zhou , Y. Wei , A. M. El‐Toni , F. Zhang , D. Zhao , J. Am. Chem. Soc. 2014, 136, 15086. 25251874 10.1021/ja508733r

[25] M. Wu , Q. Meng , Y. Chen , Y. Du , L. Zhang , Y. Li , L. Zhang , J. Shi , Adv. Mater. 2015, 27, 215. 25423915 10.1002/adma.201404256

[26] C. Yuan , T. Wu , J. Mao , T. Chen , Y. Li , M. Li , Y. Xu , B. Zeng , W. Luo , L. Yu , G. Zheng , L. Dai , J. Am. Chem. Soc. 2018, 140, 7629. 29792331 10.1021/jacs.8b03010

[27] S. A. Mian , X. Gao , S. Nagase , J. Jang , Theor. Chem. Acc. 2011, 130, 333.

[28] W. O. Yah , H. Xu , H. Soejima , W. Ma , Y. Lvov , A. Takahara , J. Am. Chem. Soc. 2012, 134, 12134. 22765271 10.1021/ja303340f

[29] C. Xue , M. Li , C. Liu , Y. Li , Y. Fei , Y. Hu , K. Cai , Y. Zhao , Z. Luo , Angew. Chem., Int. Ed. 2021, 60, 8938. 10.1002/anie.202016872 33543529

[30] P. A. Mabrouk , A. M. Orville , J. D. Lipscomb , E. I. Solomon , J. Am. Chem. Soc. 1991, 113, 4053.

[31] M. Zhang , R. X. Song , Y. Y. Liu , Z. G. Yi , X. F. Meng , J. W. Zhang , Z. M. Tang , Z. W. Yao , Y. Liu , X. G. Liu , W. B. Bu , Chem 2019, 5, 2171.

[32] I. L. Cameron , N. K. Smith , T. B. Pool , R. L. Sparks , Cancer Res. 1980, 40, 1493. 7370987

[33] I. Z. Nagy , G. Lustyik , V. Z. Nagy , B. Zarándi , C. Bertoni‐Freddari , J. Cell Biol. 1981, 90, 769. 7287822 10.1083/jcb.90.3.769 PMC2111914

[34] G. Lan , K. Ni , S. S. Veroneau , X. Feng , G. T. Nash , T. Luo , Z. Xu , W. Lin , J. Am. Chem. Soc. 2019, 141, 4204. 30779556 10.1021/jacs.8b13804

[35] T. Luo , K. Ni , A. Culbert , G. Lan , Z. Li , X. Jiang , M. Kaufmann , W. Lin , J. Am. Chem. Soc. 2020, 142, 7334. 32248686 10.1021/jacs.0c02129

[36] Y. Manevich , K. D. Held , J. E. Biaglow , Radiat. Res. 1997, 148, 580. 9399704

[37] B. Ding , J. Sheng , P. Zheng , C. Li , D. Li , Z. Cheng , P. Ma , J. Lin , Nano Lett. 2021, 21, 8281. 34591494 10.1021/acs.nanolett.1c02790

[38] Z. Zhang , Y. Zhou , S. Zhao , L. Ding , B. Chen , Y. Chen , Adv. Sci. 9, 2022, 2203583. 10.1002/advs.202203583 PMC9762308 36266982

[39] T. Bergsbaken , S. L. Fink , B. T. Cookson , Nat. Rev. Microbiol. 2009, 7, 99. 19148178 10.1038/nrmicro2070 PMC2910423

[40] Y. Wang , W. Gao , X. Shi , J. Ding , W. Liu , H. He , K. Wang , F. Shao , Nature 2017, 547, 99. 28459430 10.1038/nature22393

[41] J. Shi , Y. Zhao , K. Wang , X. Shi , Y. Wang , H. Huang , Y. Zhuang , T. Cai , F. Wang , F. Shao , Nature 2015, 526, 660. 26375003 10.1038/nature15514

[42] V. Hornung , F. Bauernfeind , A. Halle , E. O. Samstad , H. Kono , K. L. Rock , K. A. Fitzgerald , E. Latz , Nat. Immunol. 2008, 9, 847. 18604214 10.1038/ni.1631 PMC2834784

[43] C. Schorn , B. Frey , K. Lauber , C. Janko , M. Strysio , H. Keppeler , U. S. Gaipl , R. E. Voll , E. Springer , L. E. Munoz , G. Schett , M. Herrmann , J. Biol. Chem. 2011, 286, 35. 21051542 10.1074/jbc.M110.139048 PMC3012992

[44] W.‐T. He , H. Q. Wan , L. C. Hu , P. D. Chen , X. Wang , Z. Huang , Z.‐H. Yang , C.‐Q. Zhong , J. H. Han , Cell Res. 2015, 25, 1285. 26611636 10.1038/cr.2015.139 PMC4670995

[45] A. C. Reisetter , L. V. Stebounova , J. Baltrusaitis , L. Powers , A. Gupta , V. H. Grassian , M. M. Monick , J. Biol. Chem. 2011, 286, 21844. 21525001 10.1074/jbc.M111.238519 PMC3122239

[46] H. Maeda , J. Wu , T. Sawa , Y. Matsumura , K. Hori , J. Control. Release 2000, 65, 271. 10699287 10.1016/s0168-3659(99)00248-5

[47] J. Fang , H. Nakamura , H. Maeda , Adv. Drug Delivery Rev. 2011, 63, 136. 10.1016/j.addr.2010.04.009 20441782

[48] M. Shin , H. A. Lee , M. Lee , Y. Shin , J. J. Song , S. W. Kang , D. H. Nam , E. J. Jeon , M. Cho , M. Do , S. Park , M. S. Lee , J. H. Jang , S. W. Cho , K. S. Kim , H. Lee , Nat. Biomed. Eng. 2018, 2, 304. 30936449 10.1038/s41551-018-0227-9

[49] J. Xiang , Y. Zhang , X. Liu , Q. Zhou , Y. Piao , S. Shao , J. Tang , Z. Zhou , T. Xie , Y. Shen , Nano Lett. 2022, 22, 5615. 35749341 10.1021/acs.nanolett.2c02161

[50] B. A. Kairdolf , M. C. Mancini , A. M. Smith , S. Nie , Anal. Chem. 2008, 80, 3029. 18324840 10.1021/ac800068q

[51] H. Kim , S. A. Yuk , A. M. Dieterly , S. Kwon , J. Park , F. Meng , H. H. Gadalla , M. J. Cadena , L. T. Lyle , Y. Yeo , ACS Nano 2021, 15, 4576. 33645963 10.1021/acsnano.0c08694 PMC8023695

[52] M. Li , X. Sun , N. Zhang , W. Wang , Y. Yang , H. Jia , W. Liu , Adv. Sci. 2018, 5, 1800155. 10.1002/advs.201800155 PMC6051140 30027047

[53] Y. Wang , Y. Wu , K. Li , S. Shen , Z. Liu , D. Wu , Adv. Funct. Mater. 2019, 29, 1805582.

[54] Y. Li , Y. Xiao , H. P. Lin , D. Reichel , Y. Bae , E. Y. Lee , Y. Jiang , X. Huang , C. Yang , Z. Wang , Biomaterials 2019, 188, 160. 30352320 10.1016/j.biomaterials.2018.10.019

[55] K. Cheng , J. Qi , J. Zhang , H. Li , X. Ren , W. Wei , L. Meng , J. Li , Q. Li , H. Zhang , W. Deng , H. Sun , L. Mei , Biomaterials 2022, 291, 121916. 36410110 10.1016/j.biomaterials.2022.121916

[56] J.‐S. Lan , L. Liu , R.‐F. Zeng , Y.‐H. Qin , J.‐W. Hou , S.‐S. Xie , S. Yue , J. Yang , R. J. Y. Ho , Y. Ding , T. Zhang , Chem. Eng. J. 2021, 407, 127212.

[57] D. Shen , J. Yang , X. Li , L. Zhou , R. Zhang , W. Li , L. Chen , R. Wang , F. Zhang , D. Zhao , Nano Lett. 2014, 14, 923. 24467566 10.1021/nl404316v

