# Supplementary material for: Tumour‐microenvironment‐responsive Na2S2O8 nanocrystals encapsulated in hollow organosilica–metal–phenolic networks for cycling persistent tumour‐dynamic therapy
Source: Exploration (Beijing). 2023 Nov 14;4(2):20230054. doi: 10.1002/EXP.20230054 (PMC11022624; doi:10.1002/EXP.20230054)
Supplement: Supplementary file 1 — Supporting Information [file EXP2-4-20230054-s001.docx]

**Supporting Information**

**Tumor-microenvironment-responsive Na_2_S_2_O_8_ nanocrystals encapsulated in hollow organosilica-metal-phenolic networks for cycling persistent tumor-dynamic therapy**

Yang Li, ^1, 2, 3, ||^ Jinyan Lin, ^1, ||^ Yueyang He, ^4^ Kaiyuan Wang, ^5, 6^ Cailin Huang, ^2, 3^ Ruifeng Zhang, ^2, 3^ Xiaolong Liu ^1, 2, 3, *^

^1^ The United Innovation of Mengchao Hepatobiliary Technology Key Laboratory of Fujian Province, Mengchao Hepatobiliary Hospital of Fujian Medical University, Fuzhou 350025, P. R. China

^2^ CAS Key Laboratory of Design and Assembly of Functional Nanostructures, Fujian Institute of Research on the Structure of Matter, Chinese Academy of Sciences, Fuzhou 350002, P. R. China

^3^ Department of Translational Medicine & Xiamen Key Laboratory of Rare Earth Photoelectric Functional Materials, Xiamen Institute of Rare-Earth Materials, Haixi Institute, Chinese Academy of Sciences, Xiamen 361021, P. R. China

^4^ Xiang’an Hospital of Xiamen University, School of Medicine, Xiamen University, Xiamen 361100, P. R. China

^5^ Departments of Diagnostic Radiology, Surgery, Chemical and Biomolecular Engineering, and Biomedical Engineering, Yong Loo Lin School of Medicine and Faculty of Engineering, National University of Singapore, Singapore 119074, Singapore

^6^ Department of Pharmaceutics, Wuya College of Innovation, Shenyang Pharmaceutical University, Shenyang 110016, P. R. China

^||^ Yang Li and Jinyan Lin contributed equally to this work.

^*^ Corresponding authors: Xiaolong Liu, [xiaoloong.liu@gmail.com](mailto:xiaoloong.liu@gmail.com)

**
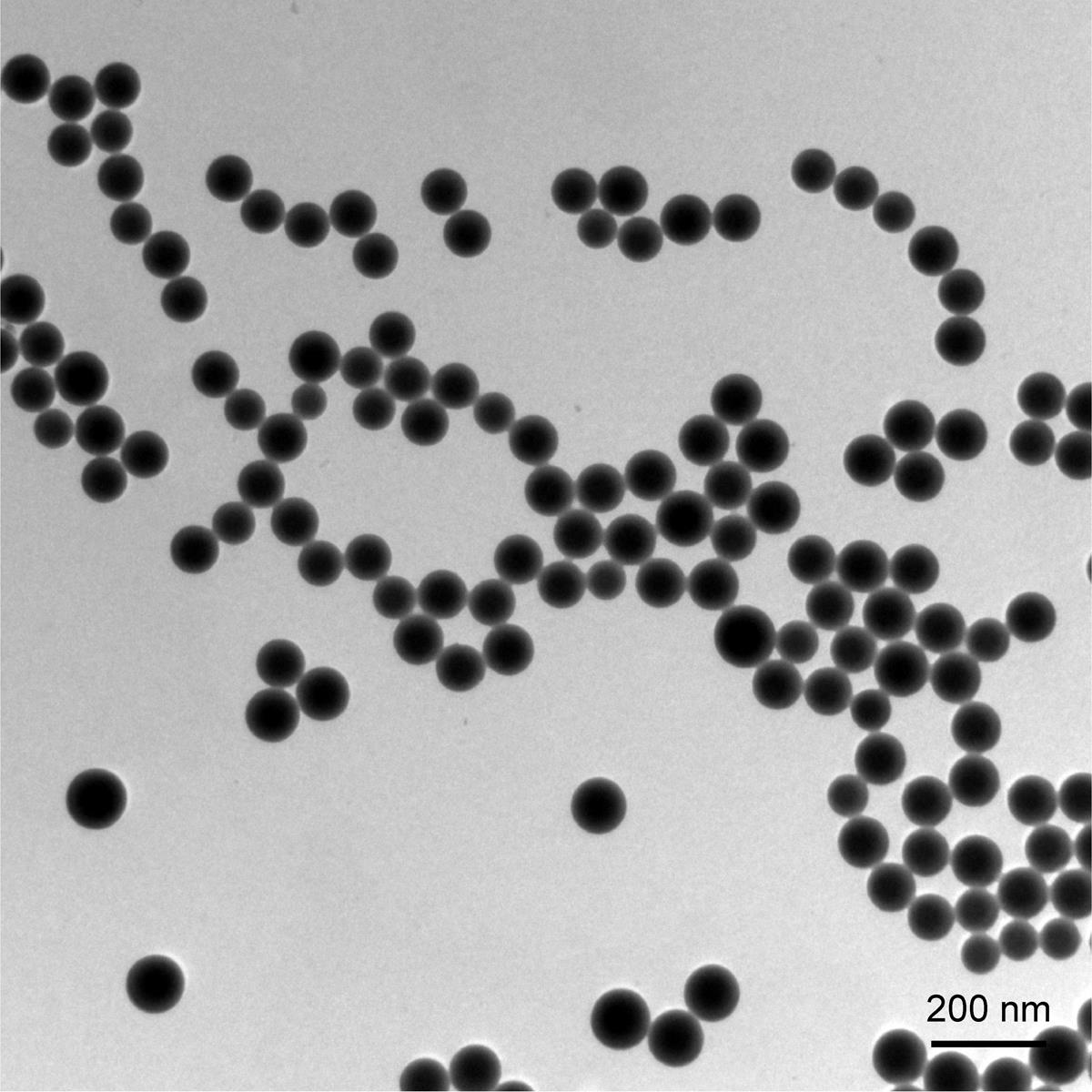
Figure S1.** TEM image of solid silica (SS) nanoparticles.


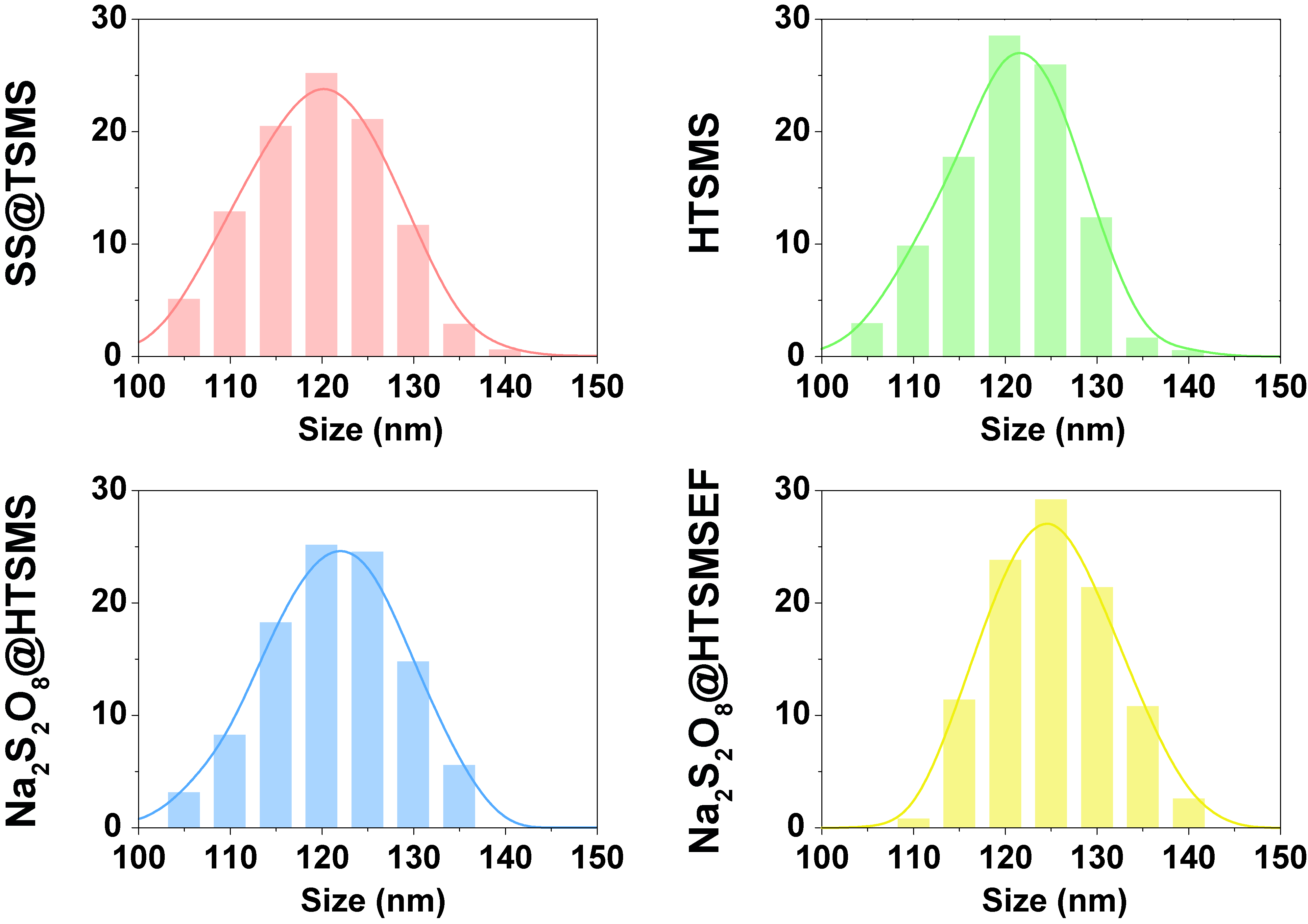


**Figure S2.** Particle size distribution of SS@TSMS, HTSMS, Na_2_S_2_O_8_@HTSMS, and Na_2_S_2_O_8_@HTSMSEF.


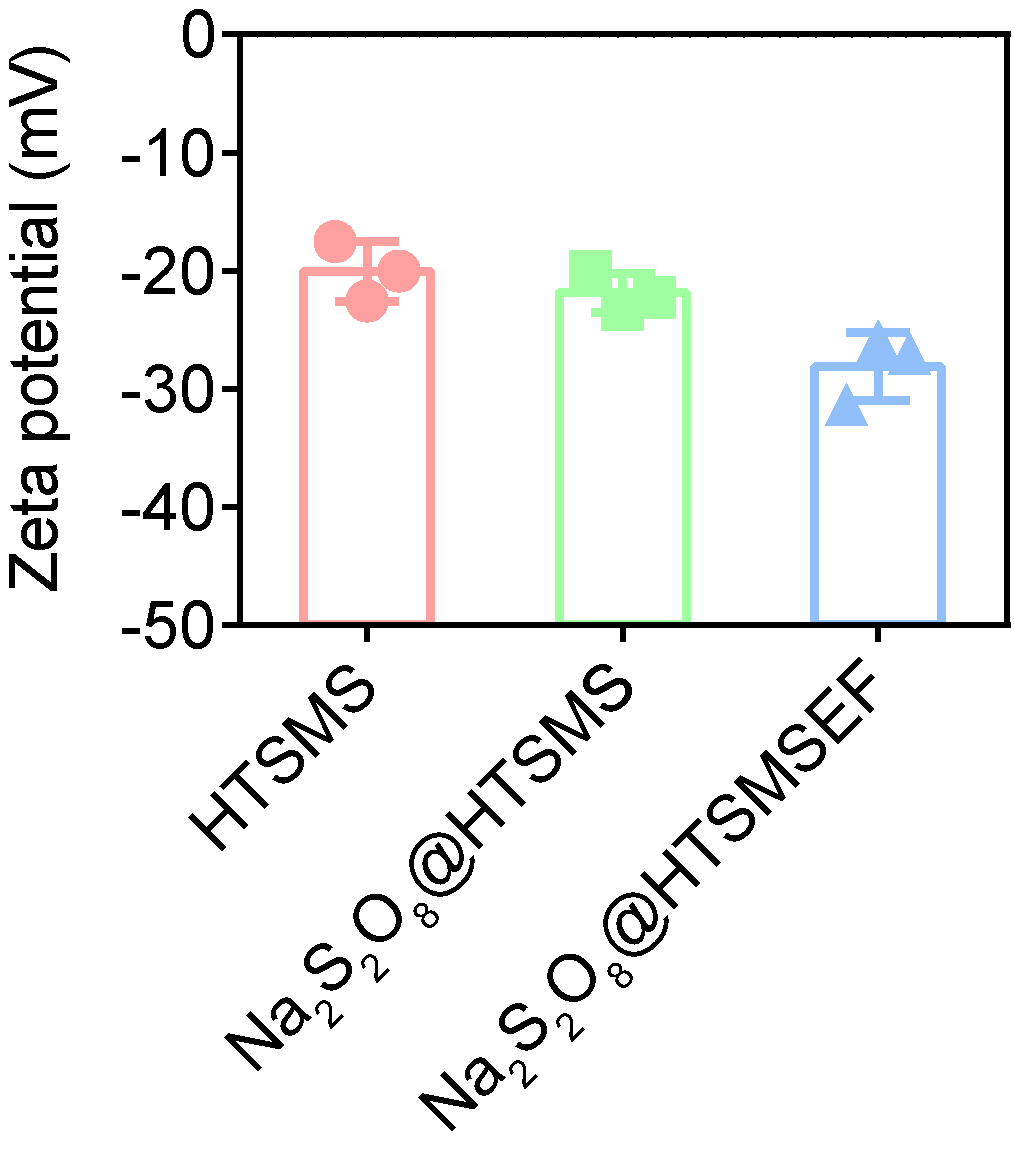


**Figure S3.** Zeta potential of HTSMS, Na_2_S_2_O_8_@HTSMS, and Na_2_S_2_O_8_@HTSMSEF.

The HTSMS and Na_2_S_2_O_8_@HTSMS presented close zeta potentials at -20.06 ± 2.53 and -21.86 ± 1.64 mV, respectively, indicating that the introduction of Na_2_S_2_O_8_ nanocrystal within the cavity of HTSMS has no significant influence on the surface charge of HTSMS. In addition, the zeta potential of Na_2_S_2_O_8_@HTSMSEF (-28.12 ± 2.85 mV) was significantly decreased as compared to that of Na_2_S_2_O_8_@HTSMS, which could be explained by the surface functionalization of EG-Fe(II) cross-linking.


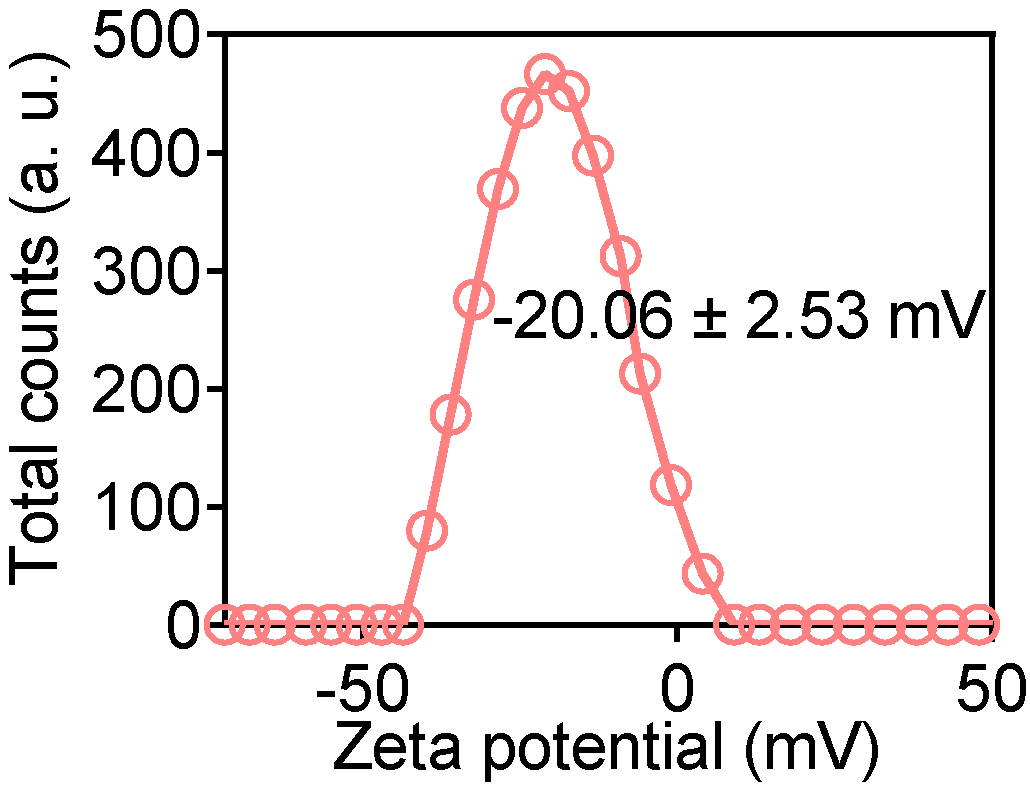


**Figure S4.** Zeta potential distribution of Na_2_S_2_O_8_@HTSMSEF dispersion.

**
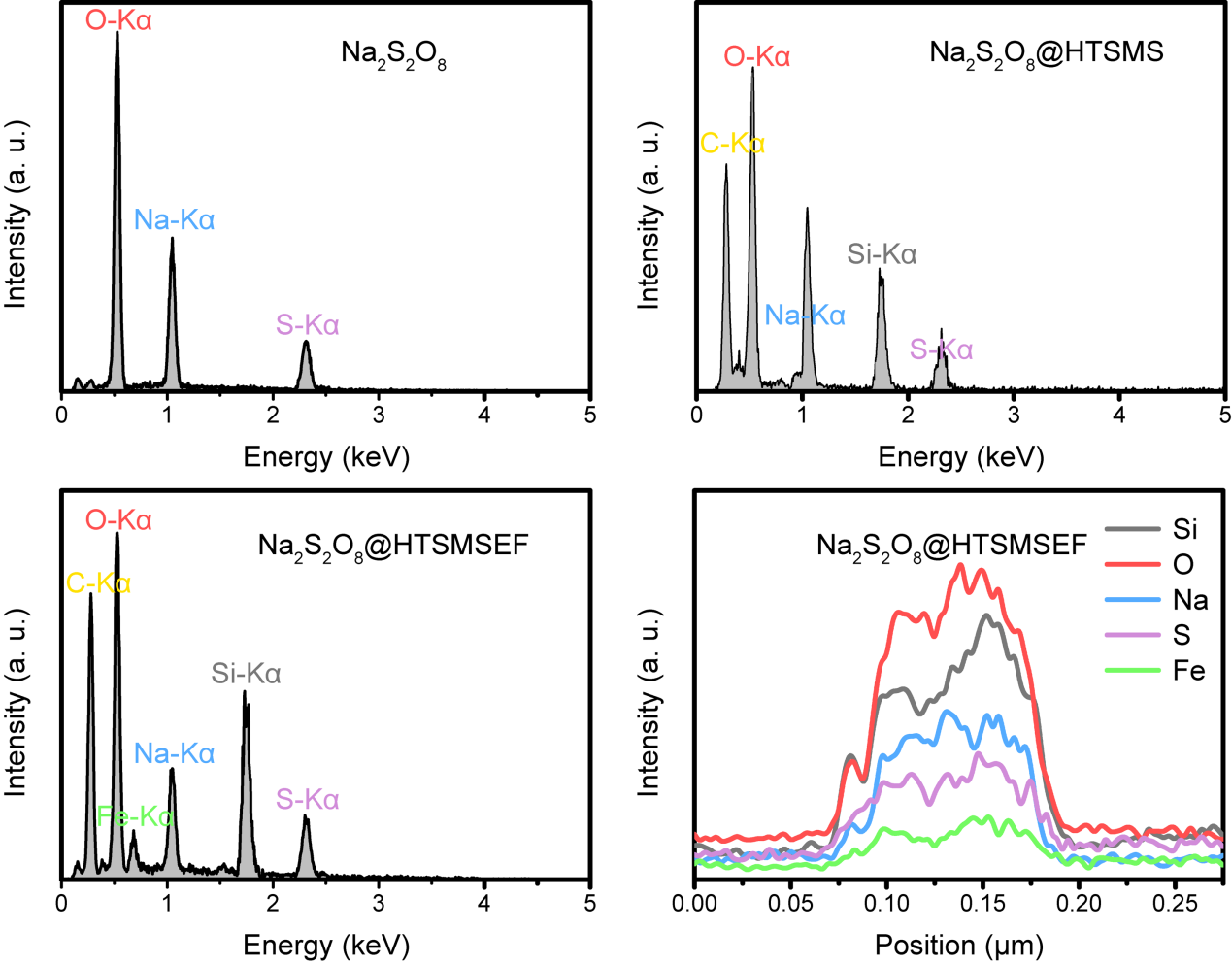
**

**Figure S5.** EDX spectra of Na_2_S_2_O_8_, Na_2_S_2_O_8_@HTSMS, and Na_2_S_2_O_8_@HTSMSEF in selected yolk-shell area.

**
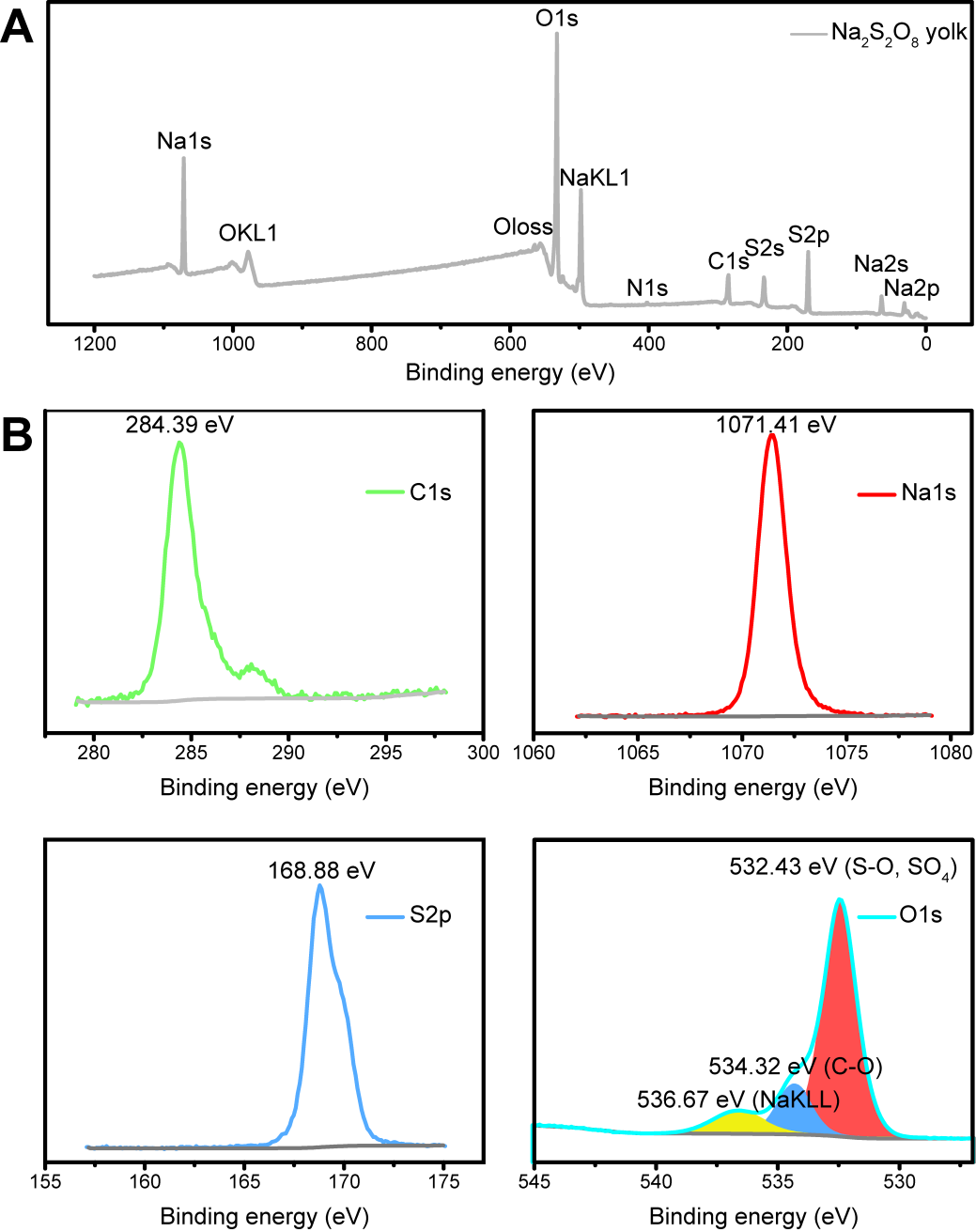
**

**Figure S6.** Original XPS spectra of wide survey scan, C1s, Na1s, S2p, and O1s region of Na_2_S_2_O_8_ nanocrystals.

**
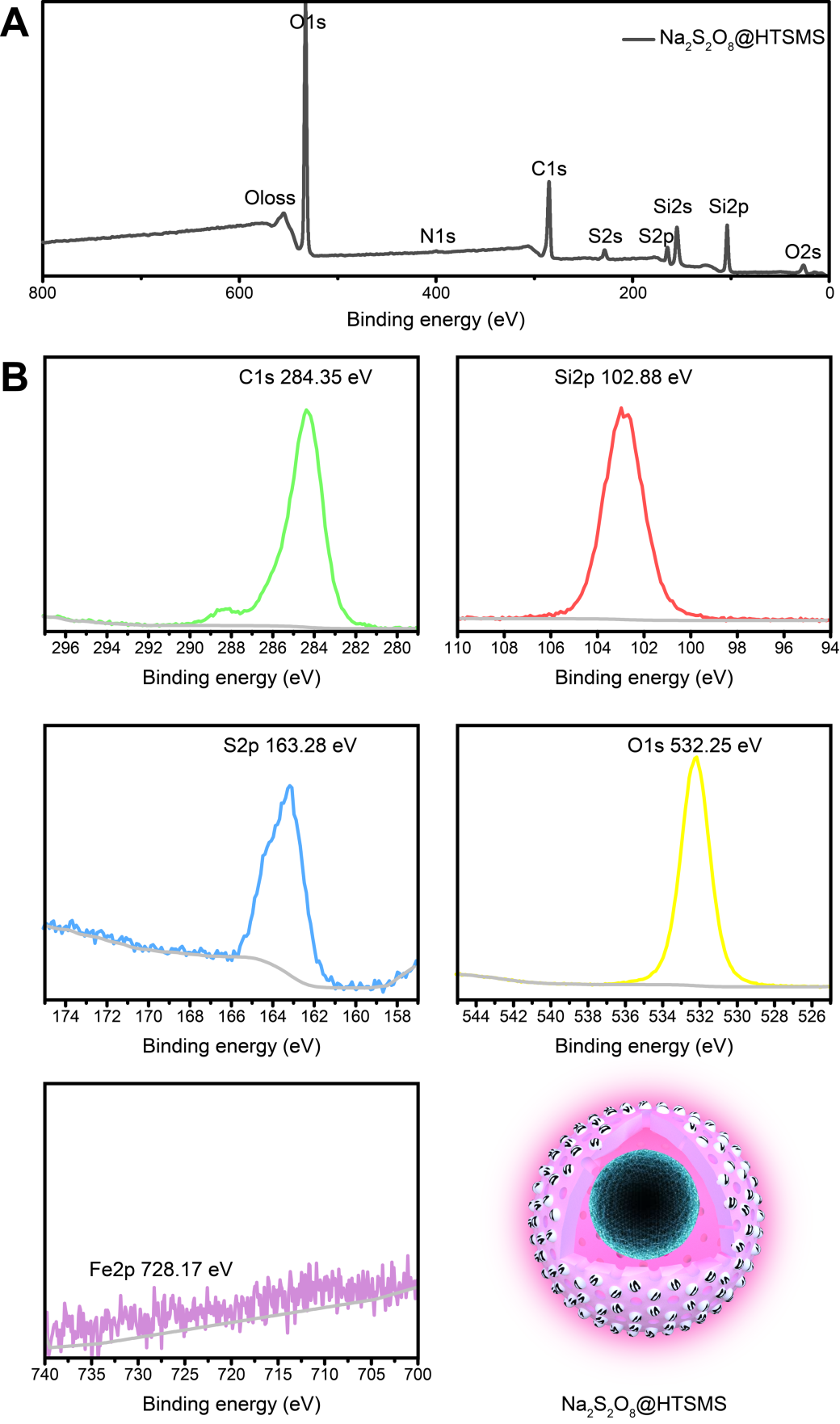
**

**Figure S7.** Original XPS spectra of wide survey scan, C1s, Si2p, S2p, O1s, and Fe2p region of Na_2_S_2_O_8_@HTSMS.


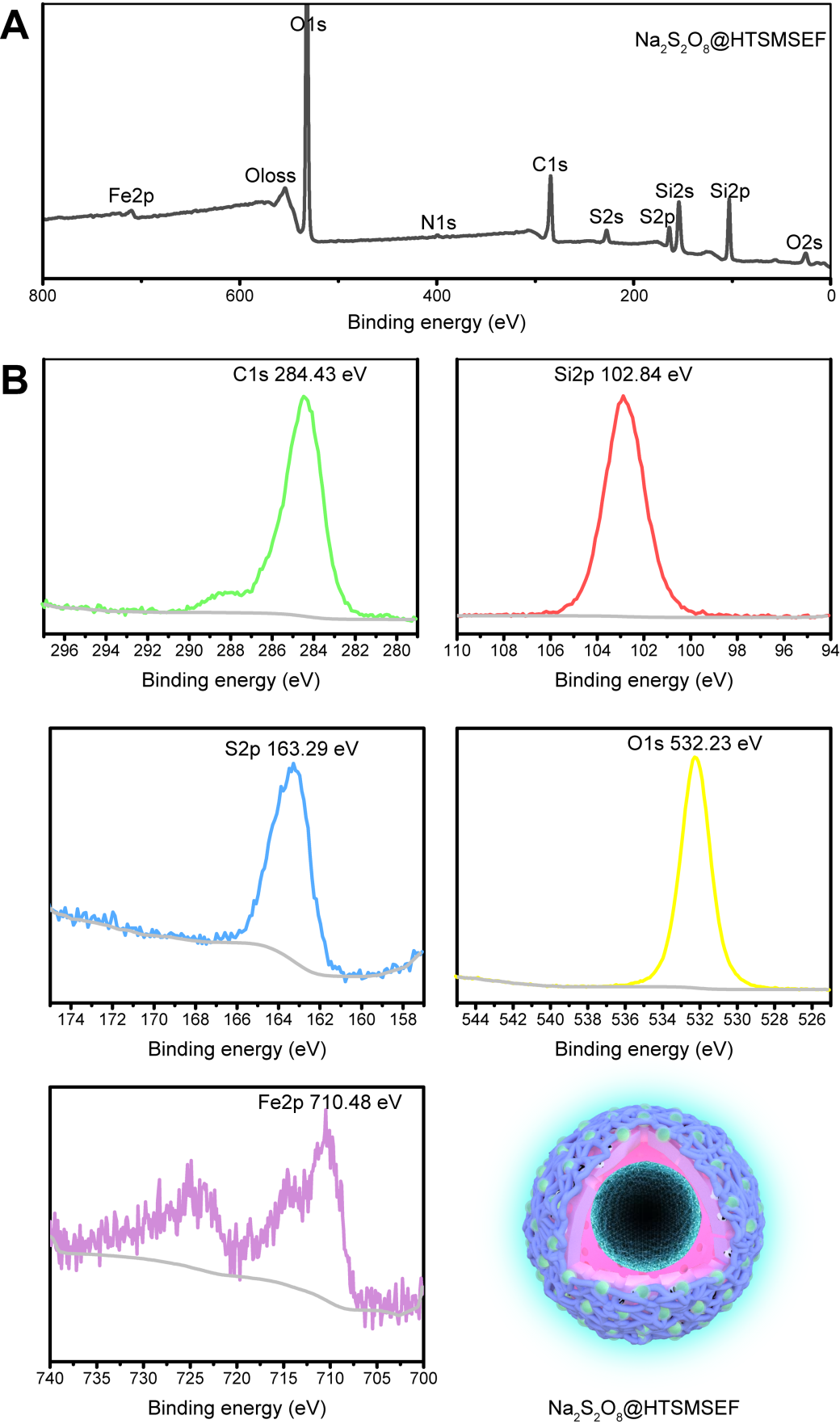


**Figure S8.** Original XPS spectra of wide survey scan, C1s, Si2p, S2p, O1s, and Fe2p region of Na_2_S_2_O_8_@HTSMSEF.


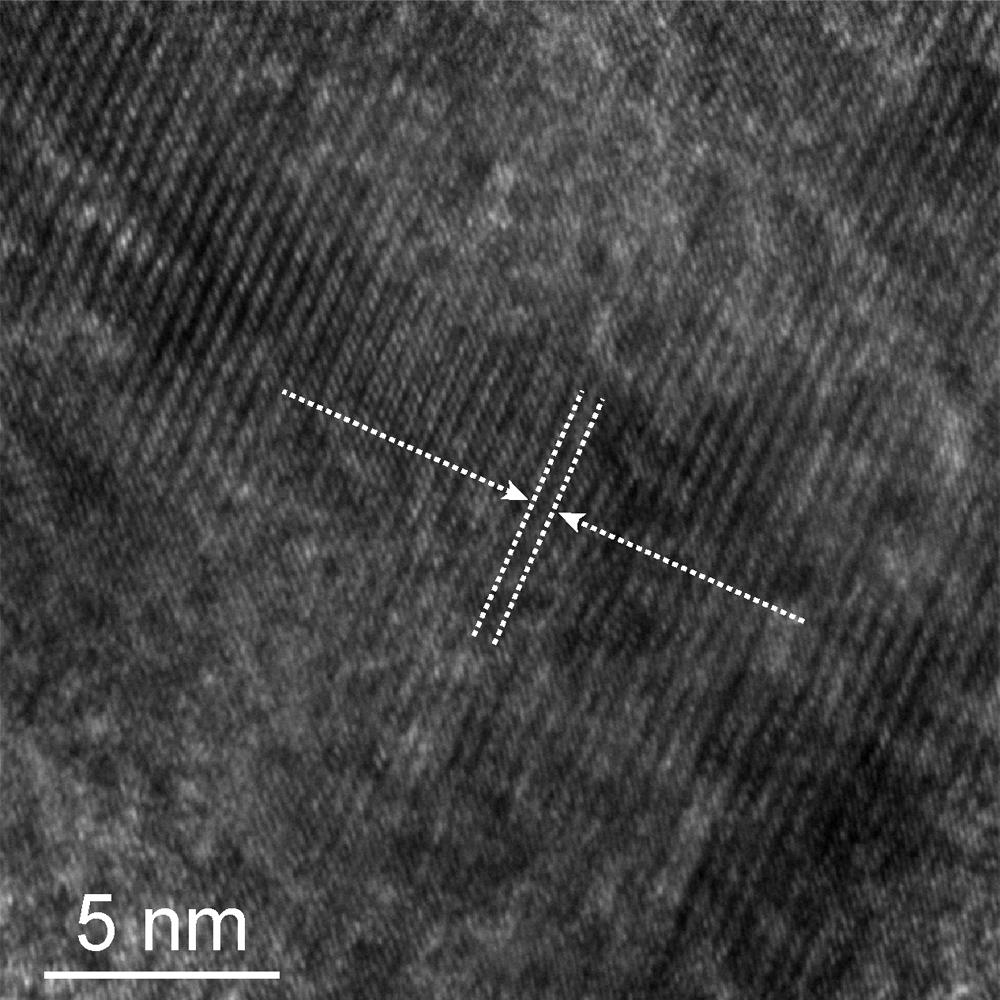


**Figure S9.** Orignial HRTEM image of Na_2_S_2_O_8_@HTSMSEF with ordered lattice fringe.

**
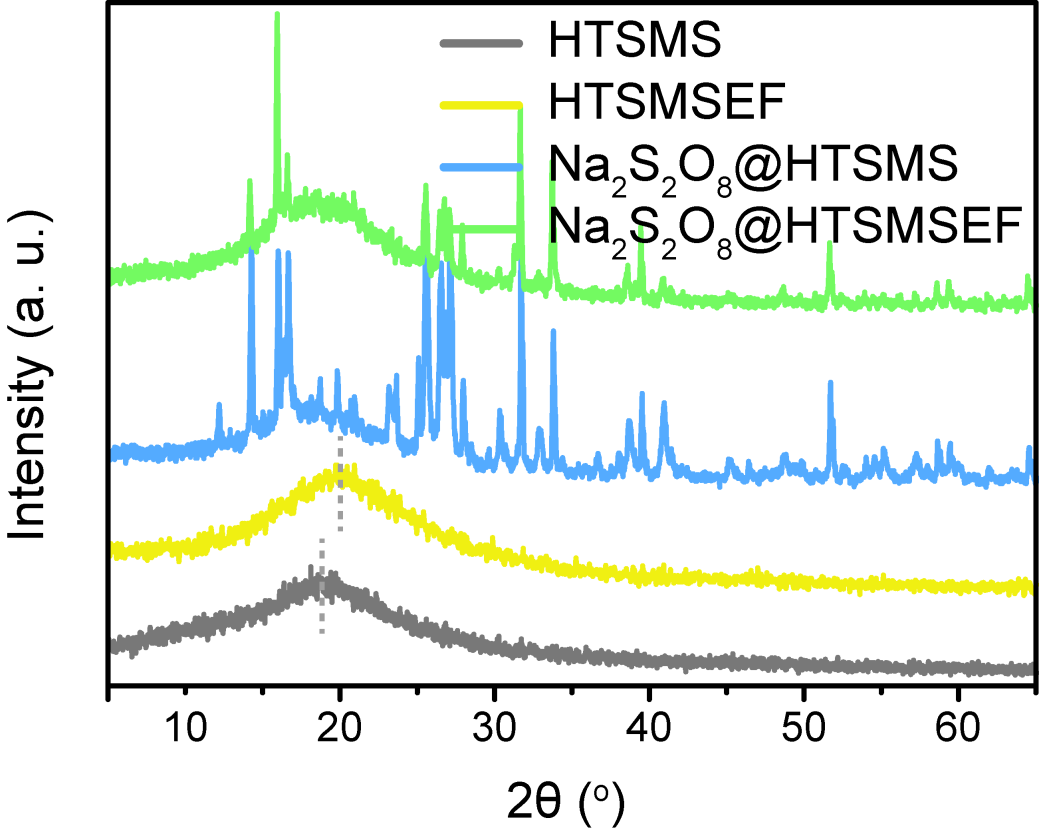
**

**Figure S10.** Original XRD pattern of HTSMS, HTSMSEF, Na_2_S_2_O_8_@HTSMS, and Na_2_S_2_O_8_@HTSMSEF.

**
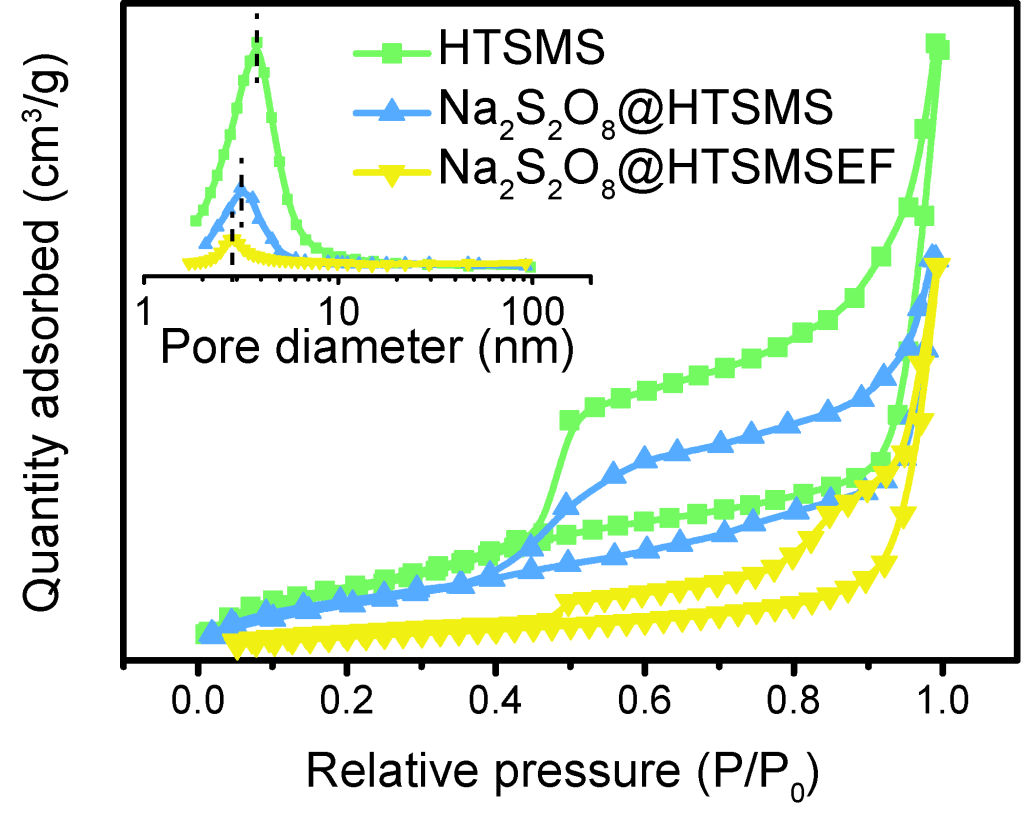
**

**Figure S11.** N_2_ adsorption-desorption isotherms and pore-size distribution curves of Na_2_S_2_O_8_@HTSMSEF. HTSMS and Na_2_S_2_O_8_@HTSMS were used as controls.


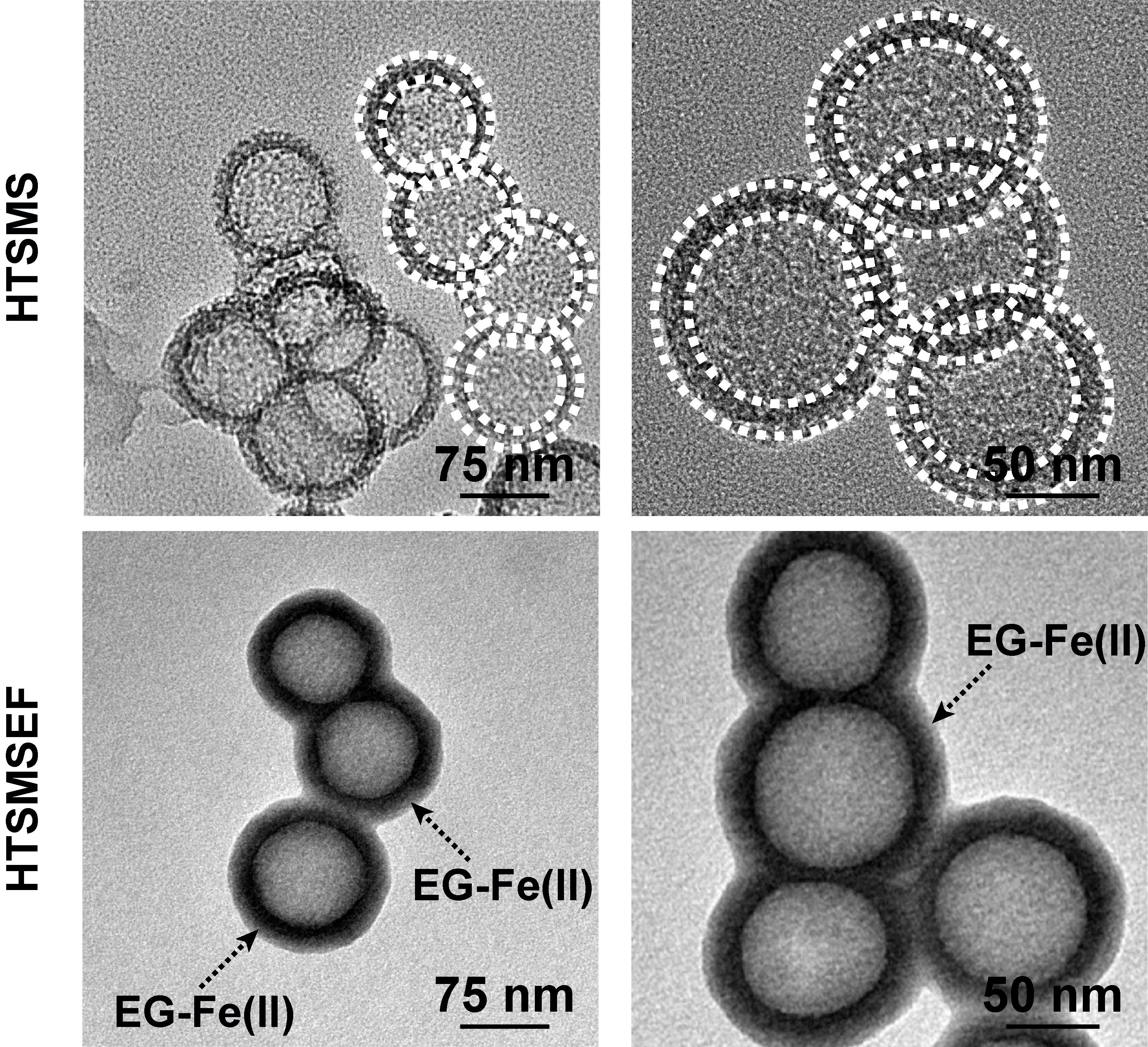


**Figure S12.** Low-magnification and high-magnification TEM images of HTSMS and HTSMSEF. The result indicated the successful functionalization of EG-Fe^2+^ coordination layer on the surface of HTSMS.

**
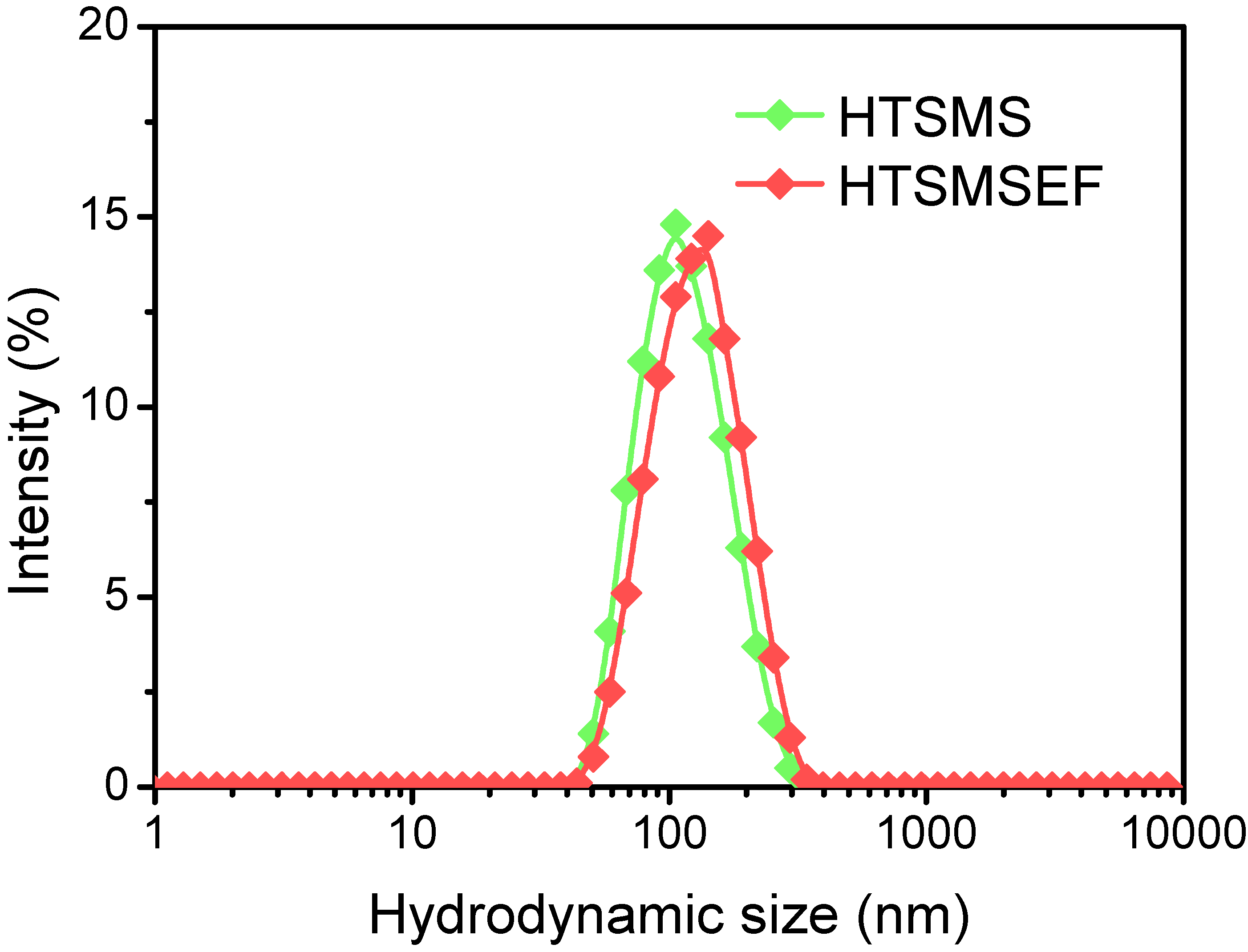
**

**Figure S13.** Hydrodynamic size distribution of HTSMS and HTSMSEF. The hydrodynamic size of HTSMSEF was slightly increased compared with that of HTSMS, indicating the surface fucntionalization of EG-Fe^2+^ coordination layer.


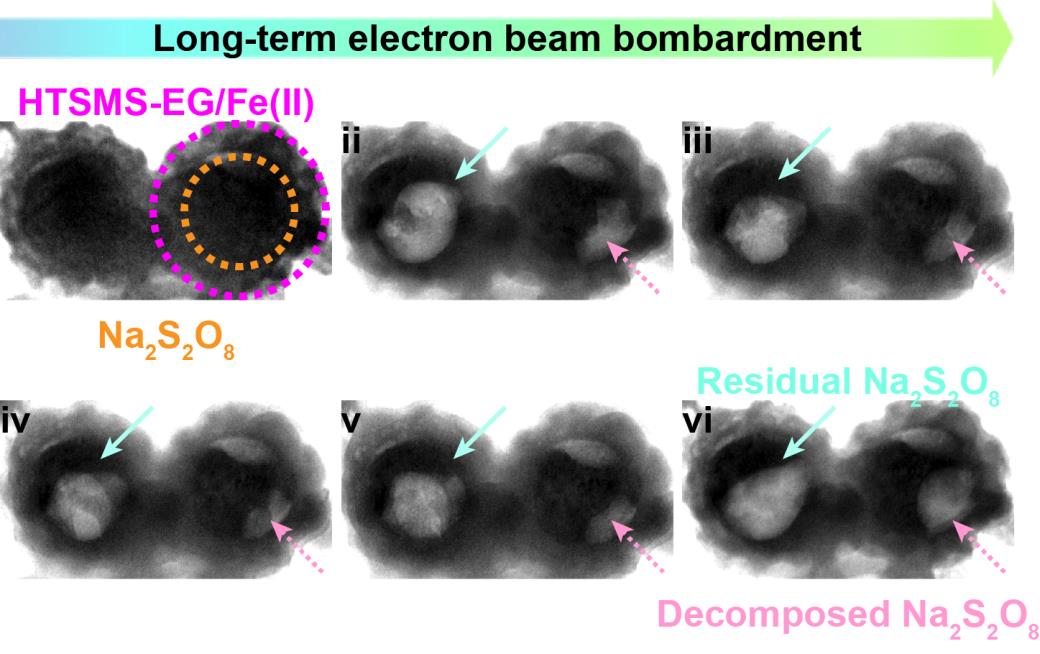


**Figure S14.** Time-lapsed TEM images of Na_2_S_2_O_8_@HTSMSEF, indicating the structural [transformation](javascript:;) of Na_2_S_2_O_8_ nanocrystals under long-term electron beam bombardment. The result indirectly proved the peculiarity of Na_2_S_2_O_8_ nanocrystals within the interior of HTSMSEF.

**
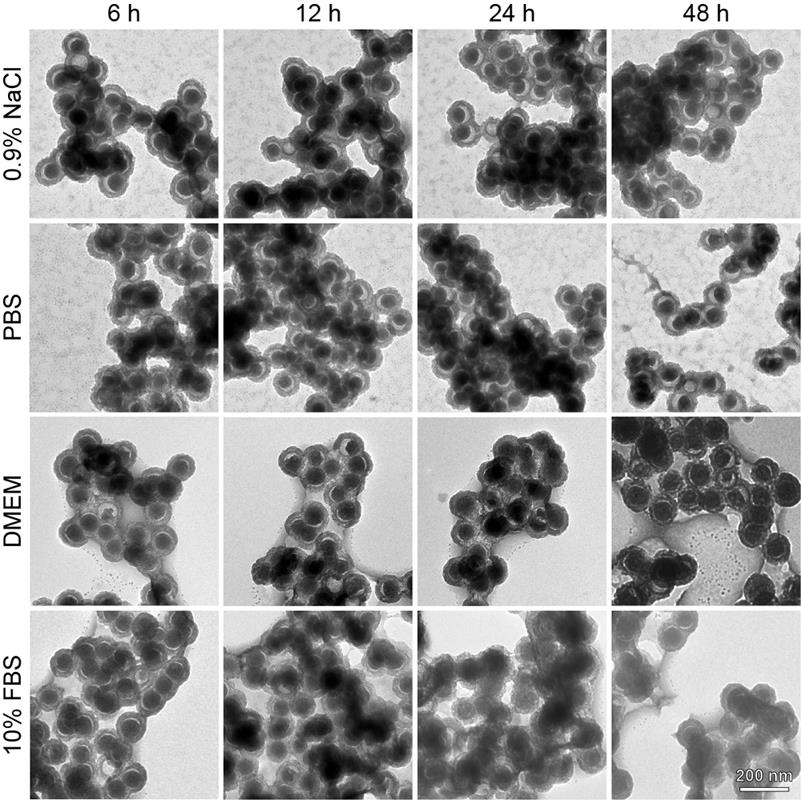
**

**Figure S15.** Physiological stability of Na_2_S_2_O_8_@HTSMSEF in 0.9% NaCl, PBS, DMEM, and 10% FBS by TEM dynamic observation.


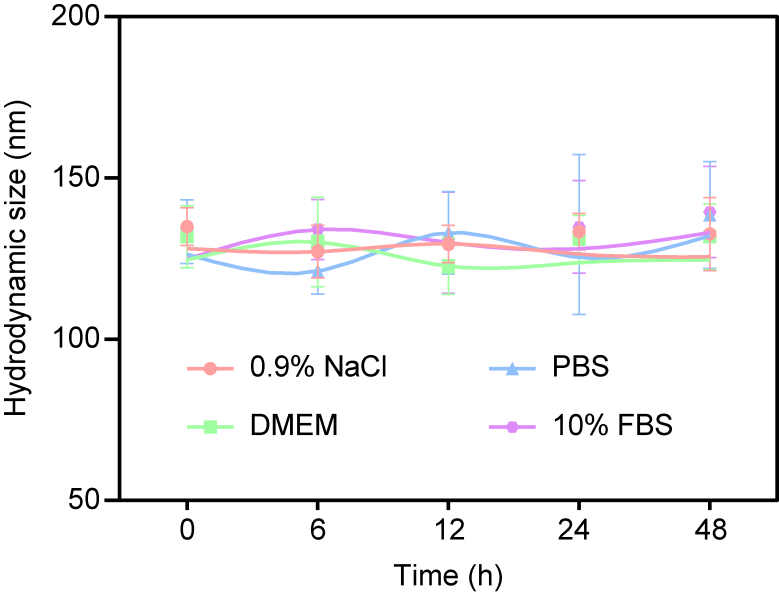


**Figure S16.** Hydrodynamic size distribution of Na_2_S_2_O_8_@HTSMSEF in 0.9% NaCl, PBS, DMEM, and 10%FBS.


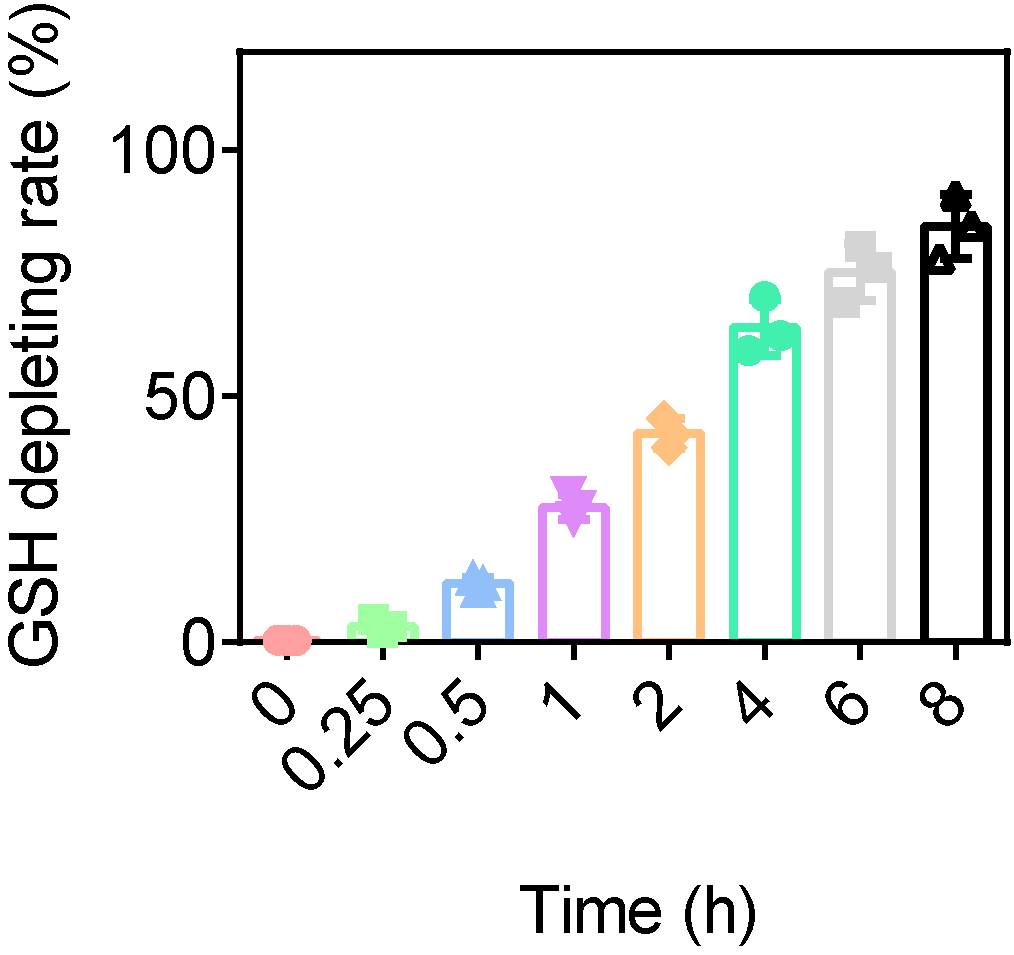


**Figure S17.** Quantity analysis regarding GSH depletion ability of Na_2_S_2_O_8_@HTSMSEF dispersion.


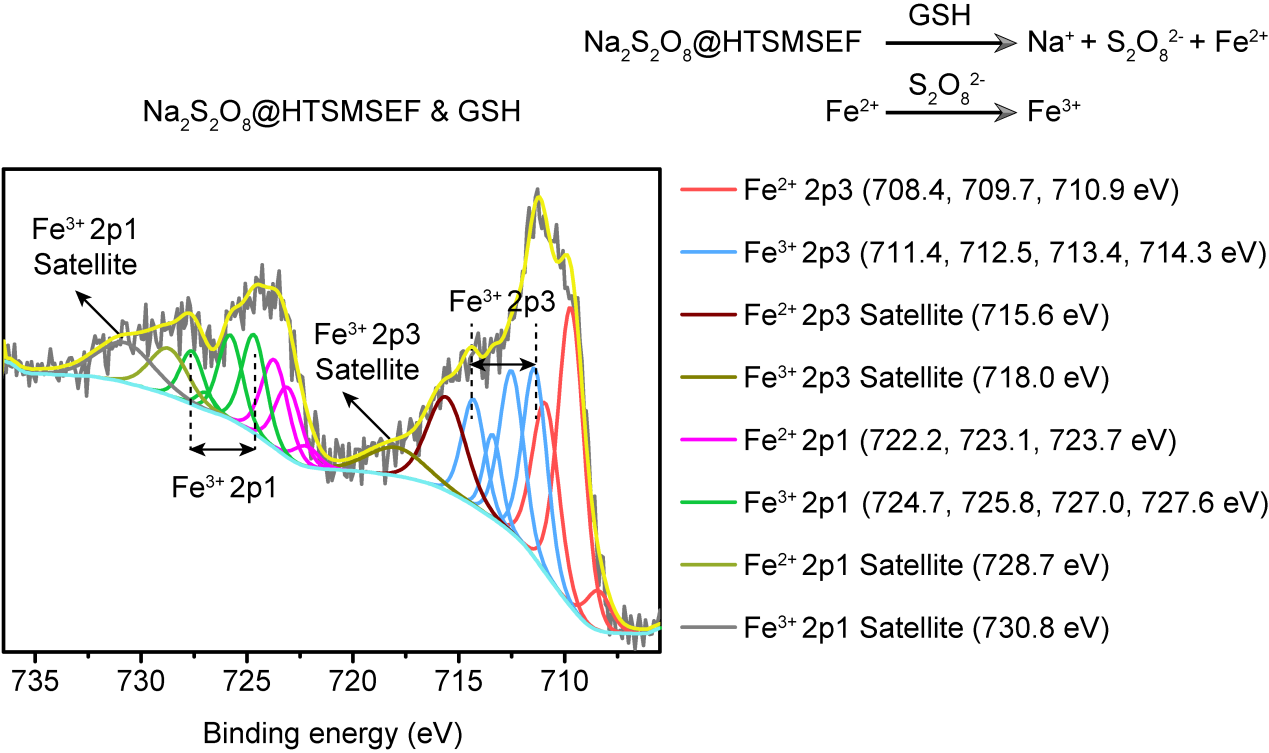


**Figure S18.** XPS spectrum of Fe2p of Na_2_S_2_O_8_@HTSMSEF after GSH treatment.

Through the XPS analysis, the Fe^3+^2p3, Fe^3+^2p1, Fe^3+^2p3 satellite, and Fe^3+^2p1 satellite peaks were newly generated in Fe2p spectra after adding GSH into Na_2_S_2_O_8_@HTSMSEF. The result not only testified the successful the GSH-triggered tetra-sulfide cleavage and shell degradation, but also implied successful release of S_2_O_8_^2−^ and subsequent transformation of Fe^2+^ into Fe^3+^ by S_2_O_8_^2−^ oxidation.


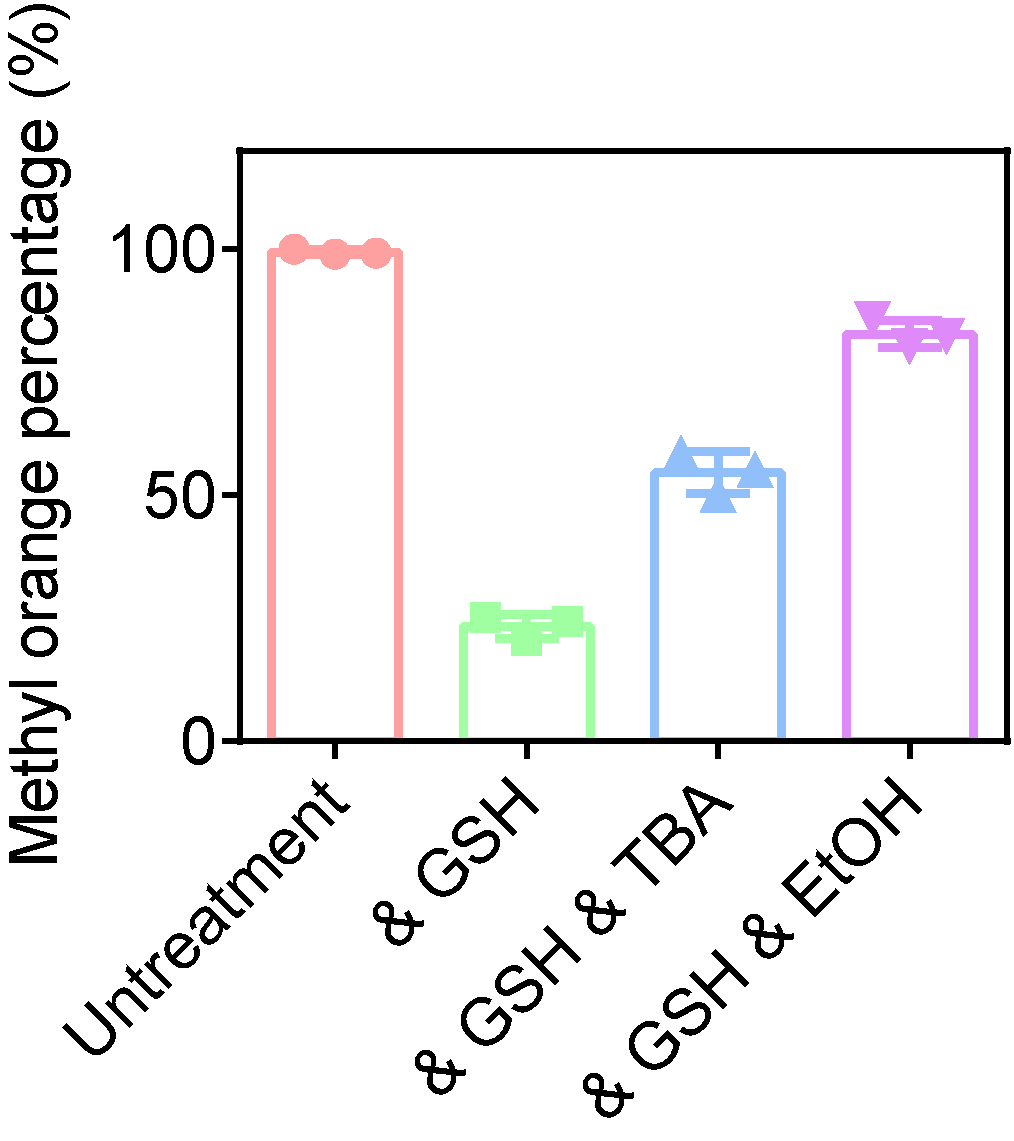


**Figure S19.** Percentage analysis of methyl orange after the addition of Na_2_S_2_O_8_@HTSMSEF pretreated with GSH and treated with ROS scavenger (TBA: •OH scavenger; EtOH: •SO_4_^−^/•OH scavenger).


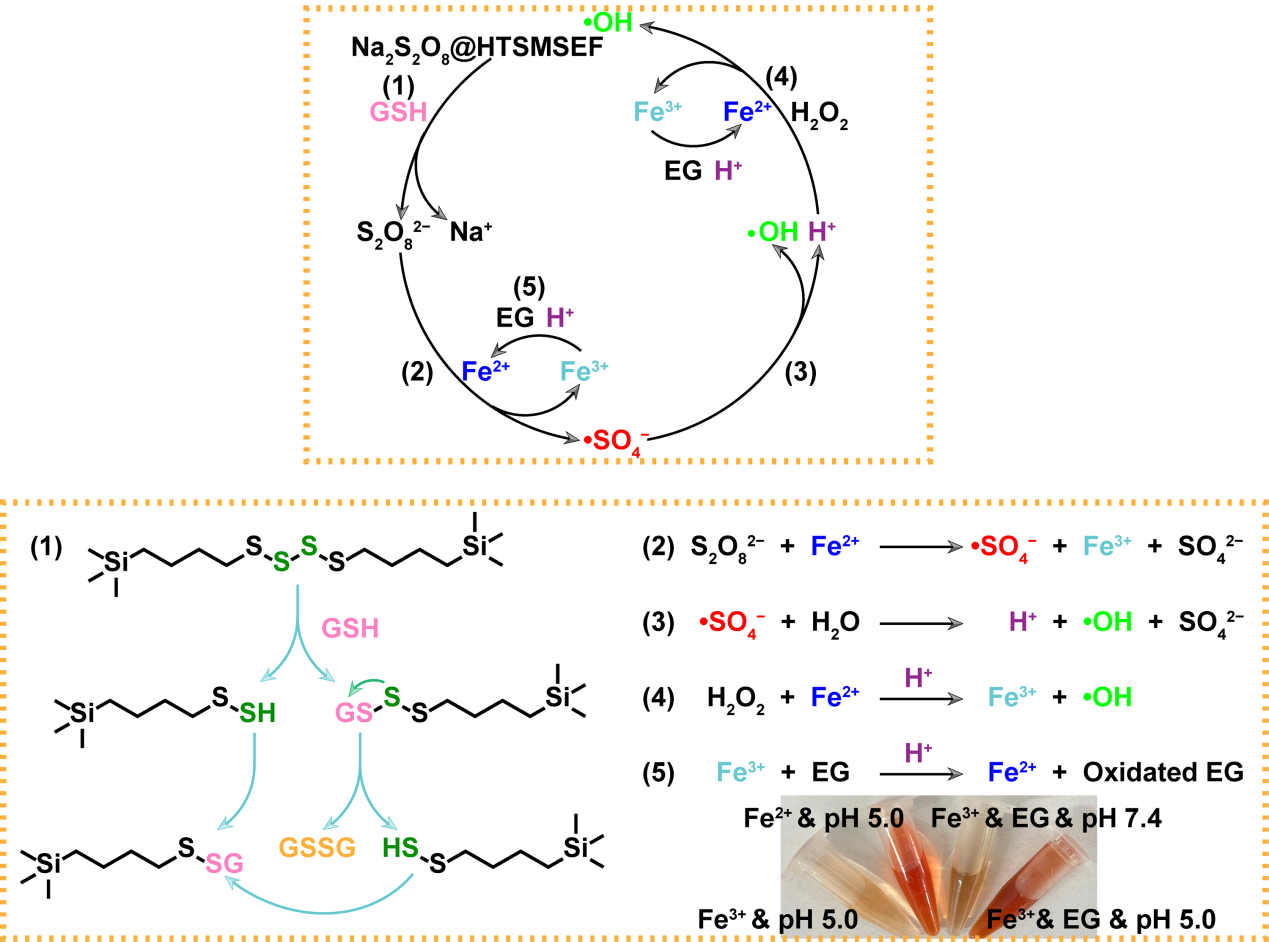


**Figure S20.** Illustration of a proposed mechanism for cascade-responsive dual cycling amplification of •SO_4_**^−^**/•OH *via* Fe^2+^-catalyzed S_2_O_8_^2−^ oxidation, •SO_4_^−^-catalyzed H_2_O decomposition, proton-intensified Fenton oxidation, and EG-activated Fe^2+^-supply-regeneration. Inset: Photographs of *o*-phenanthroline (Fe^2+^ indicator) after the addition of different formulations at different pH, indicating that the lysosomal acidity could activate EG to restore Fe^3+^ into Fe^2+^.

**
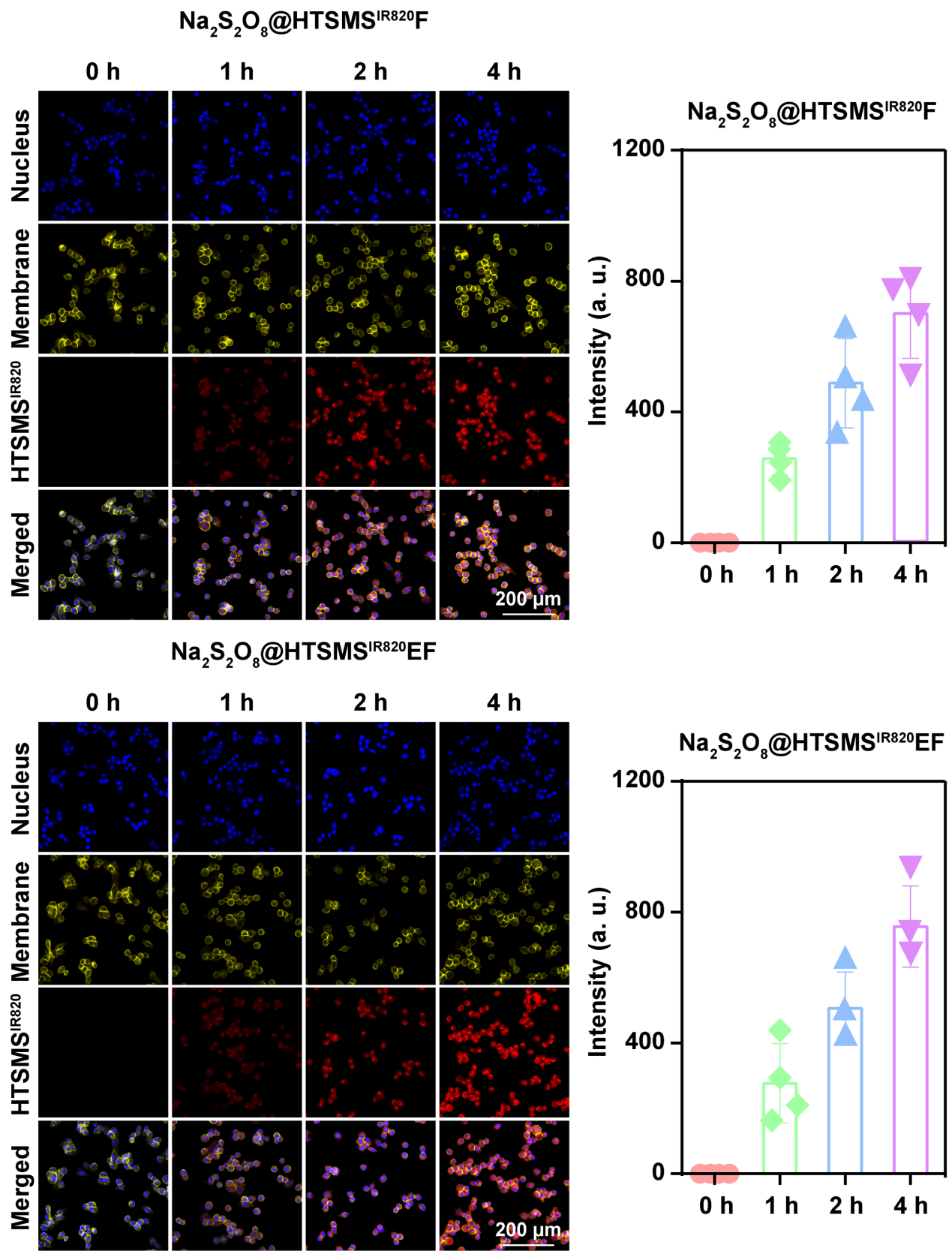
**

**Figure S21.** Confocal images and flow cytometry quantification of HepG2 cells treated with Na_2_S_2_O_8_@HTSMS^IR820^F and Na_2_S_2_O_8_@HTSMS^IR820^EF for 1, 2, and 4 h and stained with DAPI (labels the nucleus)/rhodamine-wheat germ agglutinin (labels the plasma membrane). Before investigating the cell uptake behaviors of nanoreactors, the amine functional groups of HTSMS were labeled by IR820-NHS.


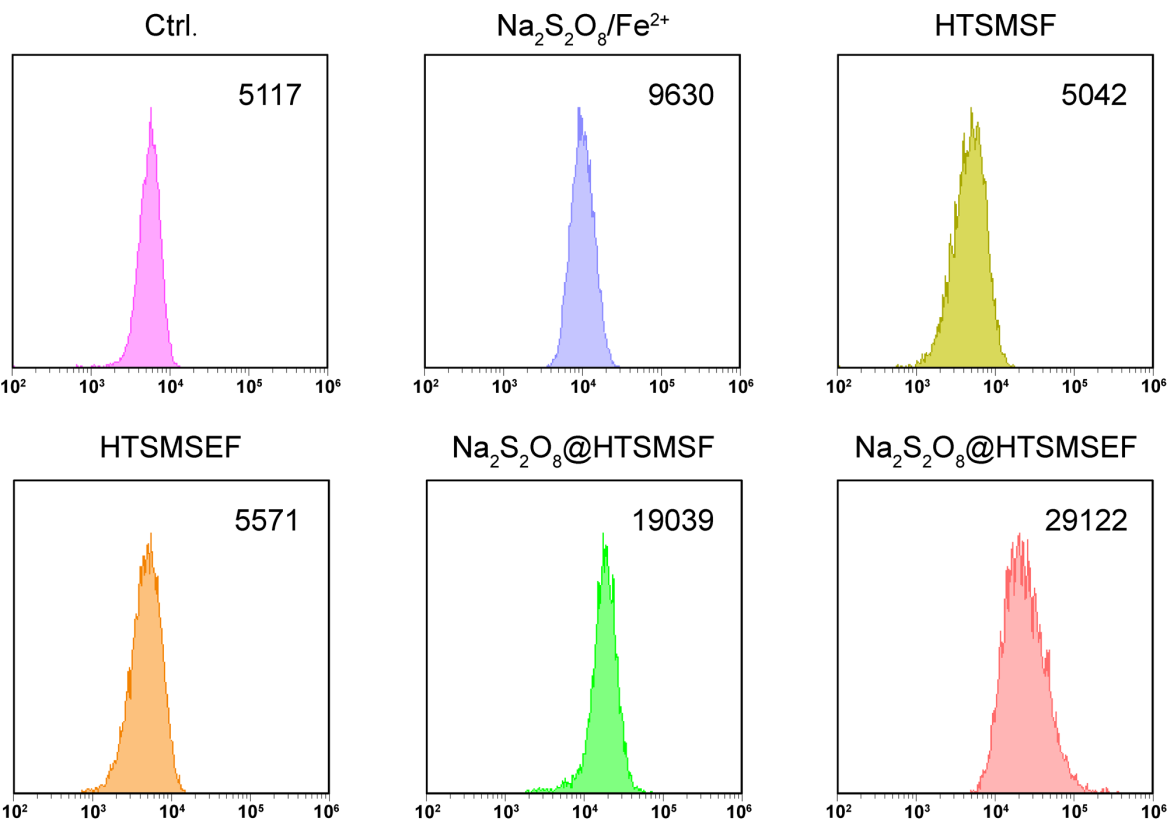


**Figure S22.** Na^+^ overload quantification of HepG2 cells incubated with different formulations for 4 h and then stained with SBFI (Na^+^ indicator) by using flow cytometry.


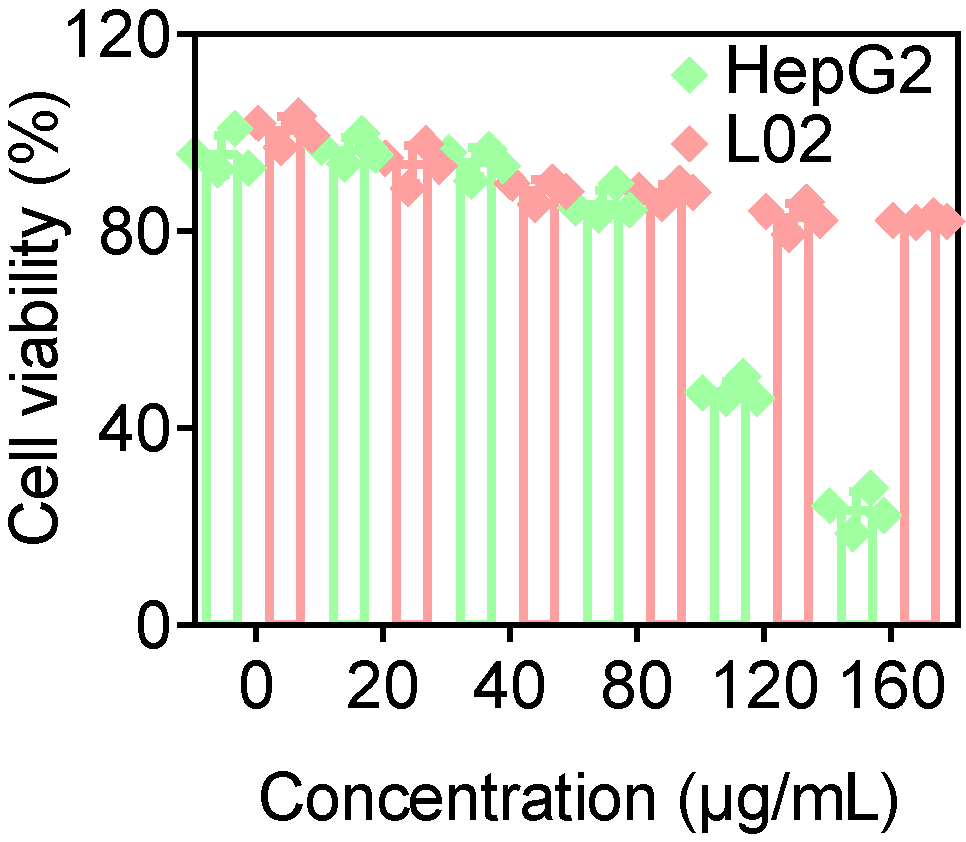


**Figure S23.** Cell viability of HepG2 and L02 cells treated with Na_2_S_2_O_8_/Fe^2+^ for 24 h.


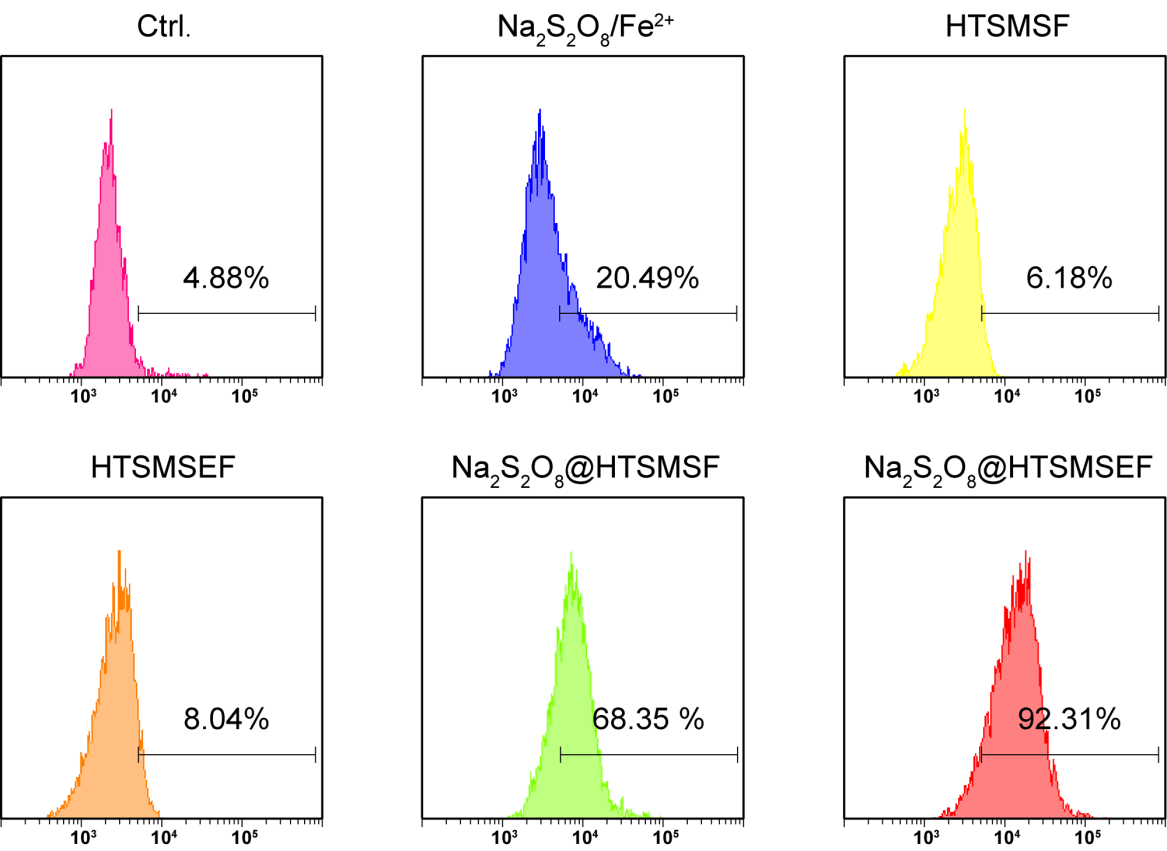


**Figure S24.** Total ROS generation quantification of HepG2 cells incubated with different formulations for 8 h and then stained with DCFH-DA by using flow cytometry.


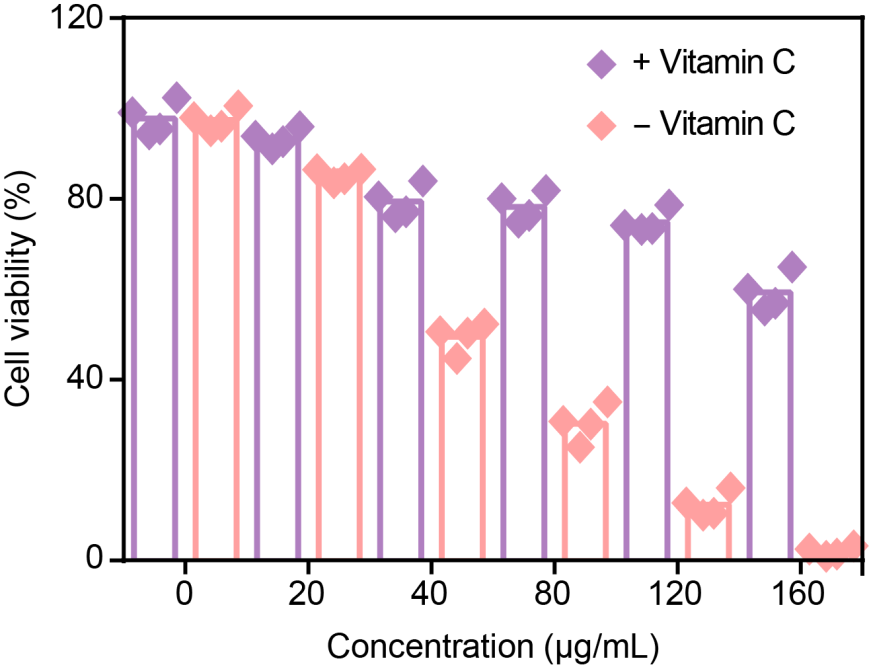


**Figure S25.** Cell viability of HepG2 cells pretreated with vitamin C and treated with Na_2_S_2_O_8_@HTSMSEF at the Na_2_S_2_O_8_ concentration from 20 to 160 μg/mL for 24 h incubation.


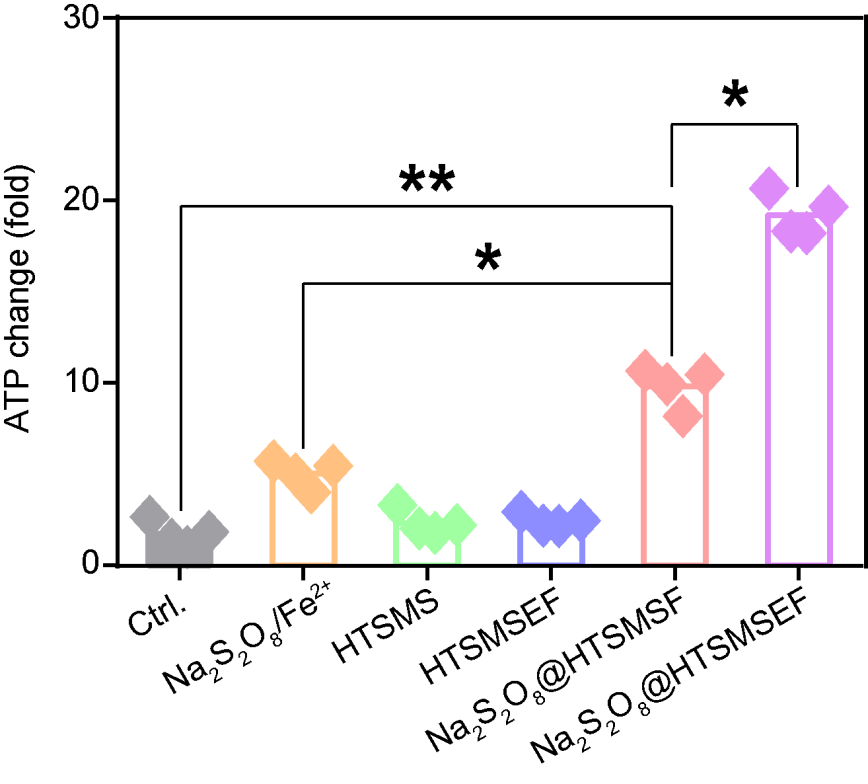


**Figure S26.** ATP change quantification of HepG2 cells incubated with different formulations.


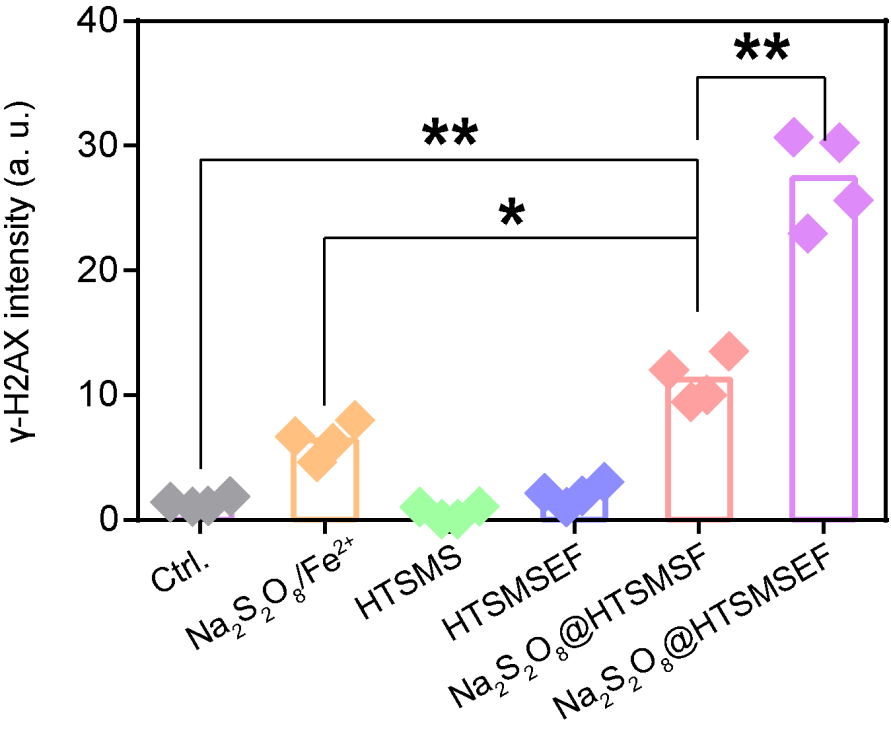


**Figure S27**. DNA double-strand breakage quantification of HepG2 cells incubated with different formulations.


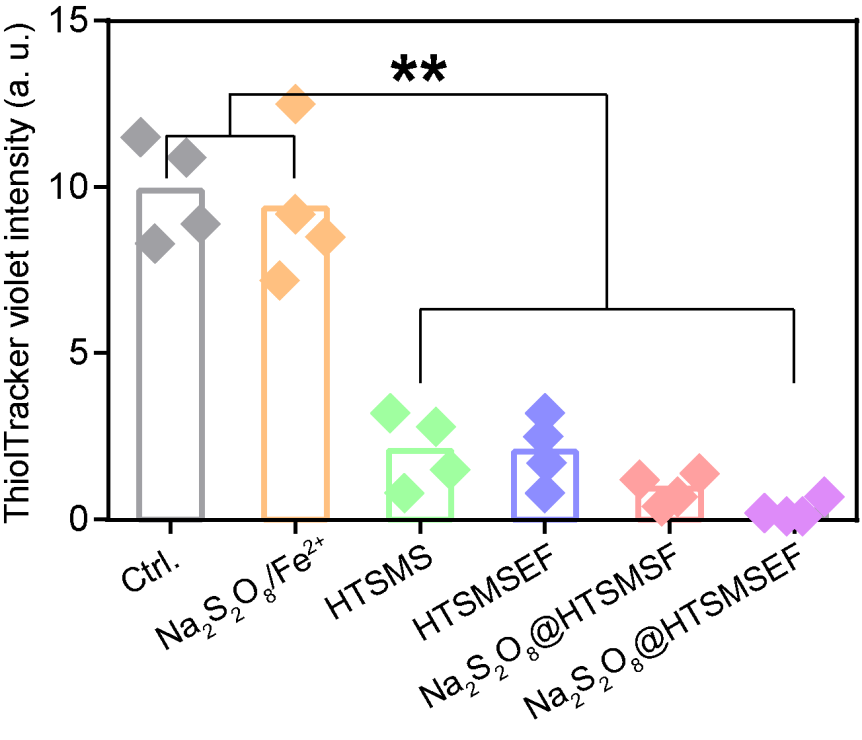


**Figure S28.** GSH exhaustion quantification of HepG2 cells incubated with different formulations.


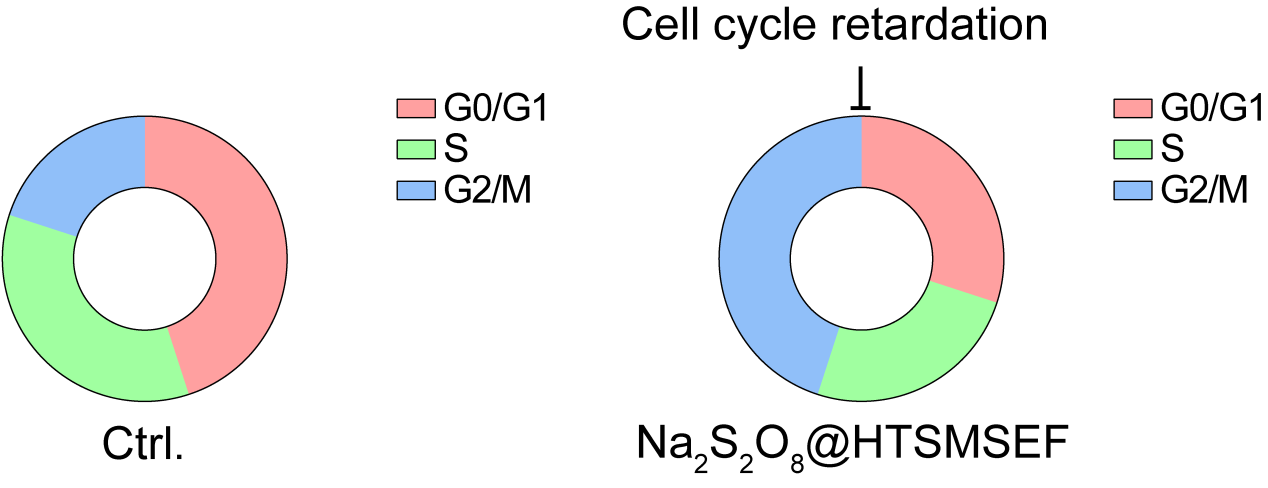


**Figure S29.** Cell cycle analysis of HepG2 cells incubated with Na_2_S_2_O_8_@HTSMSEF for 8 h using flow cytometry.


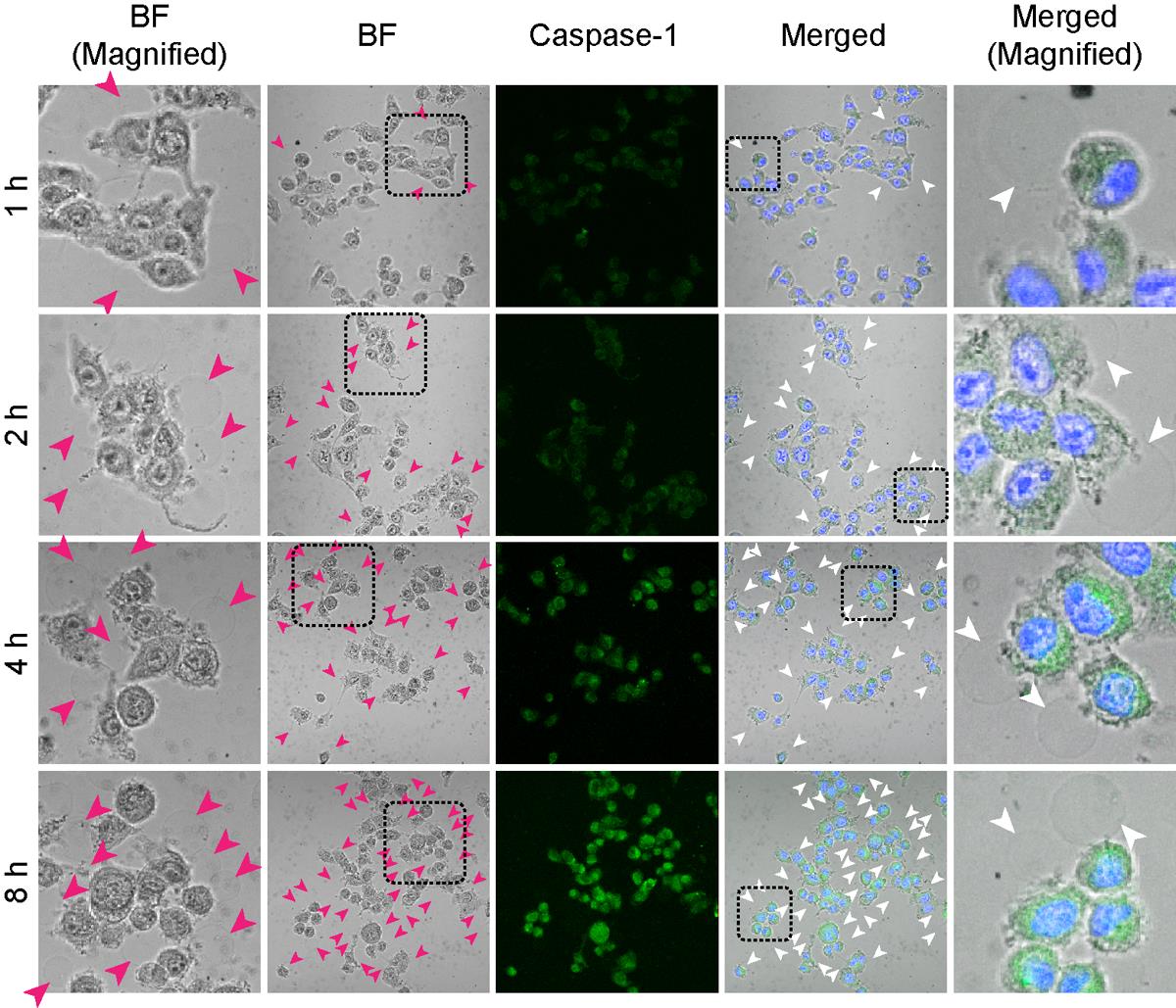


**Figure S30.** Original data regarding time-lapsed confocal observation of caspase-1 expression in HepG2 cells after treatment of Na_2_S_2_O_8_@HTSMSEF using immunoﬂuorescence. The arrows indicate the ballooning bubbles.


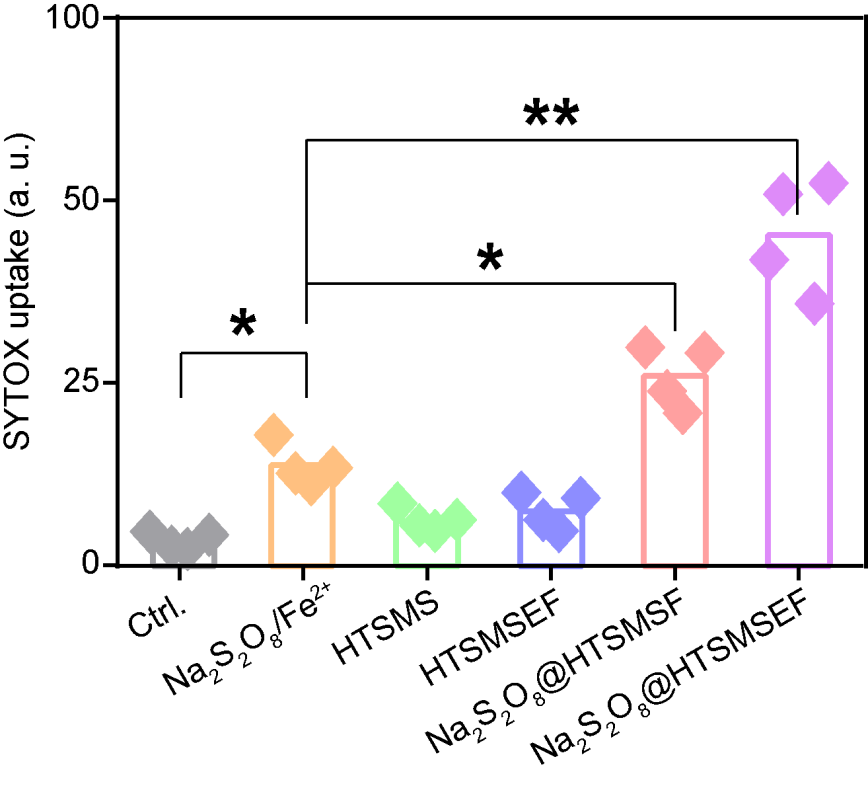


**Figure S31.** Intracellular SYTOX (a nucleic acid probe, which could only pass across the damaged plasma membrane) fluorescence intensity quantification of HepG2 cells incubated with different formulations.

Both the SYTOX green staining and PI staining demonstrated remarkably increased entry of SYTOX green and PI into HepG2 cells after the Na_2_S_2_O_8_@HTSMSEF treatment, verifying the cell membrane damage and extraordinary programmed cell death.


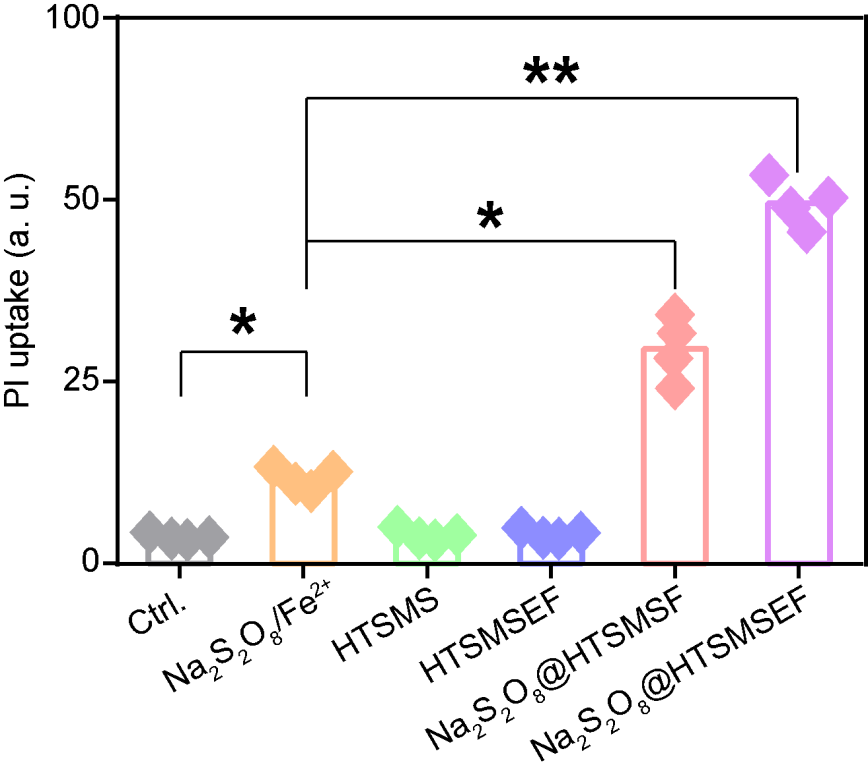


**Figure S32.** Intracellular PI fluorescence intensity quantification of HepG2 cells incubated with different formulations.


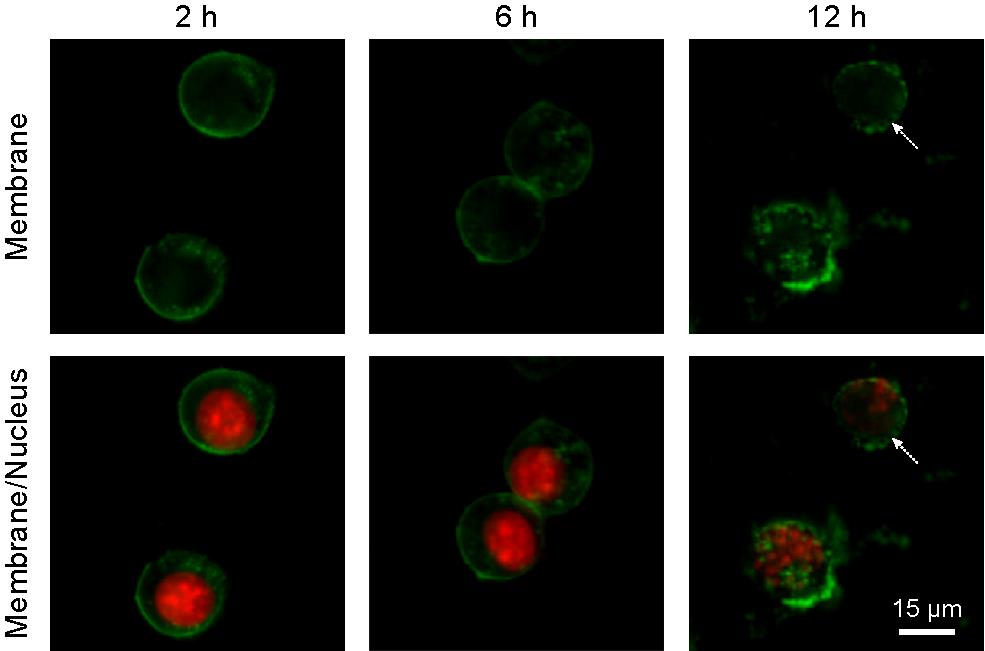


**Figure S33.** Confocal observation of cell membrane damage with pyroptosis-mediated pore formation of HepG2 cells incubated with Na_2_S_2_O_8_@HTSMSEF.

**
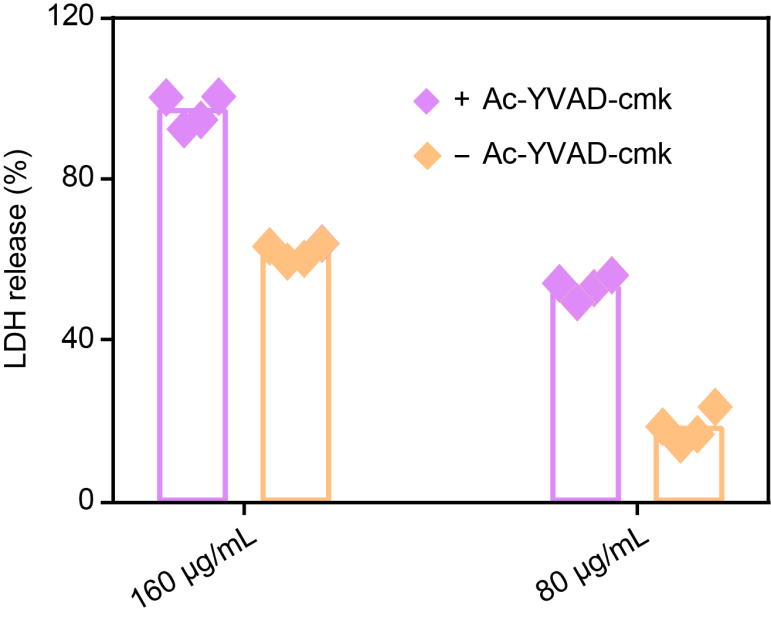
**

**Figure S34.** LDH release quantification of HepG2 cells using LDH release assay kit after pretreatment of Ac-YVAD-cmk (a capsase-1 inhibitor) and then treatment with Na_2_S_2_O_8_@HTSMSEF.


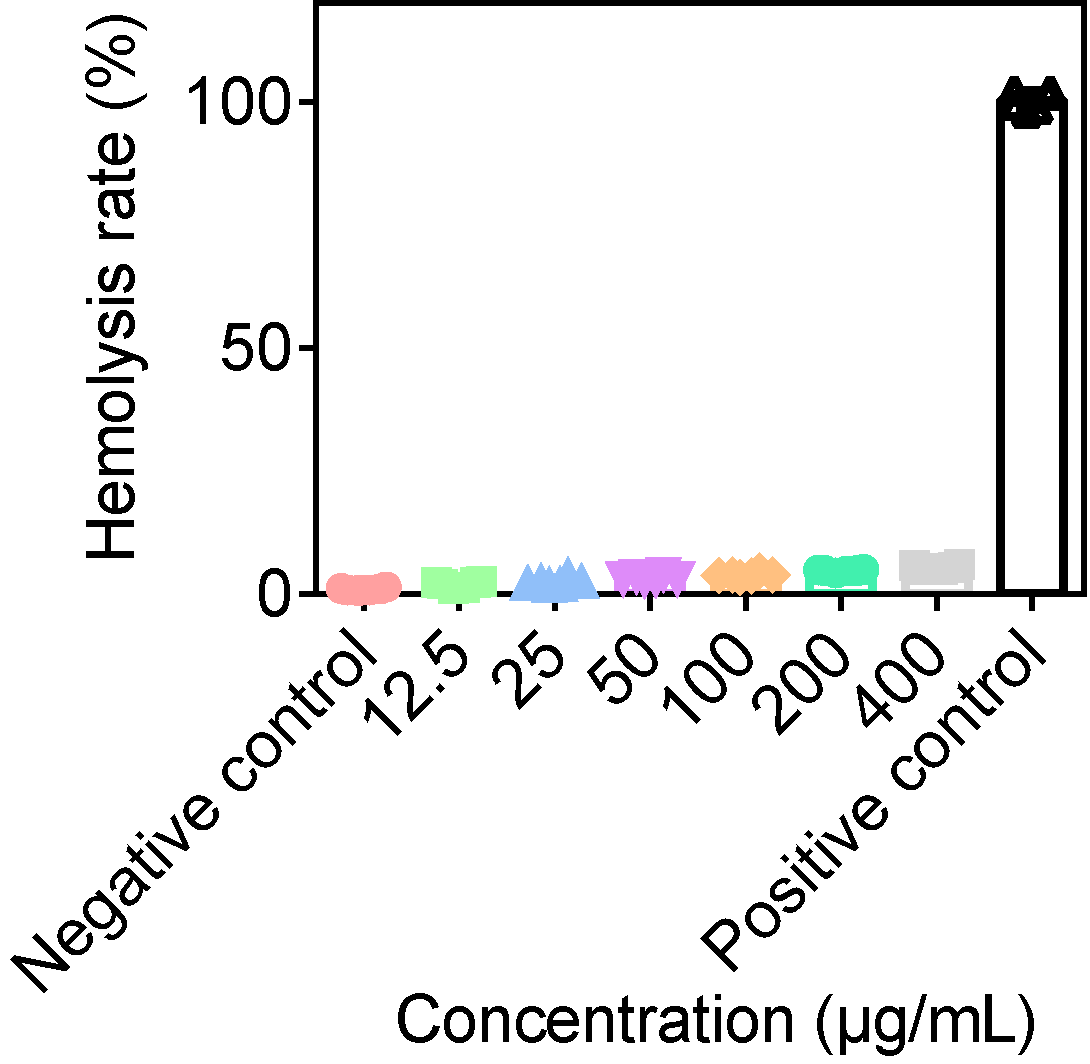


**Figure S35.** Hemolysis rate of Na_2_S_2_O_8_@HTSMSEF with different concentrations ranging from 12.5 μg/mL to 400 μg/mL.

To estimate the hemocompatibility of Na_2_S_2_O_8_@HTSMSEF, the hemolysis assay was performed. As shown in Figure SX, the Na_2_S_2_O_8_@HTSMSEF displayed no significant damage to red cell membranes even at the concentration of 400 μg/mL.


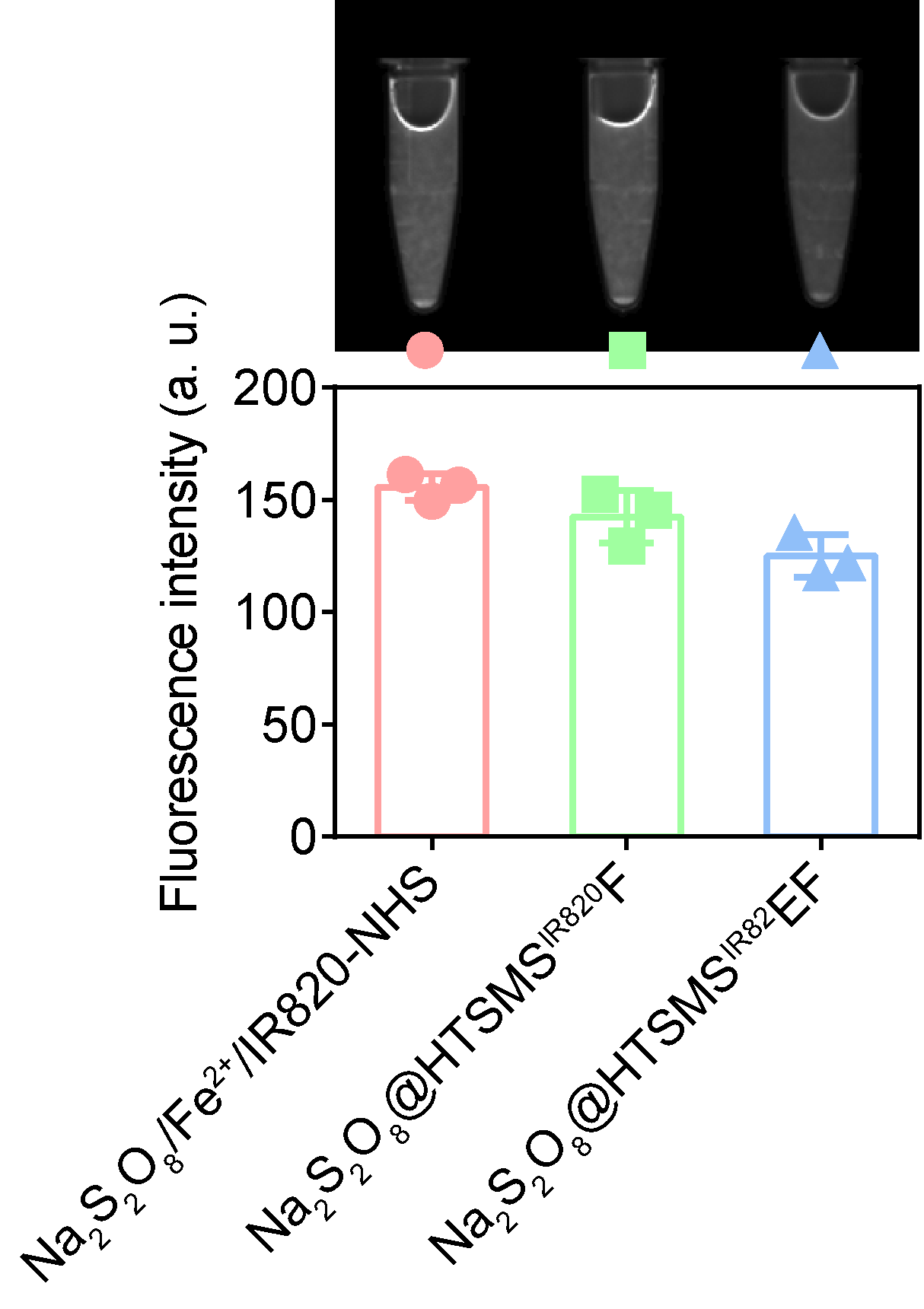


**Figure S36.** *In vitro* fluorescence signal diagram of Na_2_S_2_O_8_/Fe^2+^/IR820-NHS, Na_2_S_2_O_8_@HTSMS^IR820^F, and Na_2_S_2_O_8_@HTSMS^IR820^EF.


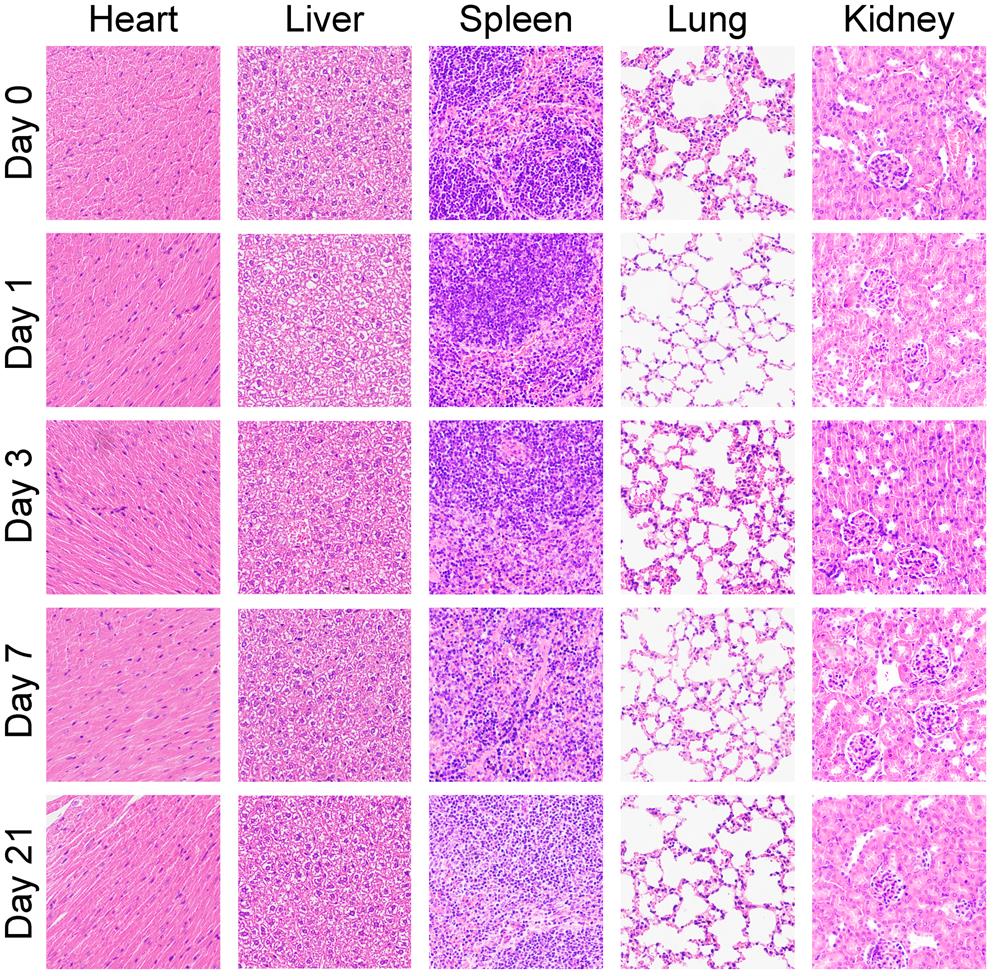


**Figure S37.** H&E staining in normal tissue slices from tumor-bearing nude mice intravenously injected with Na_2_S_2_O_8_@HTSMSEF for different time periods.
